# Supplementary material for: Fast multi-source nanophotonic simulations using augmented partial factorization
Source: Nat Comput Sci. 2022 Dec 15;2(12):815–22. doi: 10.1038/s43588-022-00370-6 (PMC10768640; doi:10.1038/s43588-022-00370-6)
Supplement: Supplementary file 1 — Supplementary Sects. 1–16, Figs. 1–11 and Table 1. [file 43588_2022_370_MOESM1_ESM.pdf]

nature computational science

Article

https://doi.org/10.1038/s43588-022-00370-6

# Fast multi-source nanophotonic simulations using augmented partial factorization

In the format provided by the authors and unedited

## CONTENTS


1. Scattering matrix 2
   A. Channels in homogeneous space 2
   B. Scattering matrix 3
   C. Fisher–Lee relation 4

2. Finite-difference discretization 5
   A. Channels in discrete homogeneous space 6
   B. Finite-difference Fisher–Lee relation 7

3. Utilizing symmetry in augmented partial factorization (APF) 8

4. Computing time and memory scaling of APF 9

5. APF with compressed input/output matrices (APF-c) 9

6. APF pseudocode 12

7. System-size scaling for disordered media simulations 13

8. Round-off error of APF 14

9. APF for very large scattering matrices 14

10. Metalens design and simulation 15

11. System-size scaling for metasurface simulations with RCWA and RGF 16

12. Angular spectrum propagation 17

13. Metalens transmission efficiency and Strehl ratio 18

14. Locally periodic approximations 18

15. APF-c compression error 19

16. Discretization error 19

References 21

## 1. SCATTERING MATRIX

In this section, we define the flux-normalized scattering matrix and derive its expression. For concreteness, here we consider the 2D transverse-magnetic fields in a two-sided system, with outgoing boundary condition in the longitudinal direction $x$ and Bloch periodic boundary condition in the transverse direction $y$. Defining the corresponding expressions for other boundary conditions and in 3D is straightforward, and the concepts apply similarly to other geometries.

We start with time-harmonic electromagnetic waves at frequency $\omega$ with $e^{-i\omega t}$ dependence on time $t$, governed by the frequency-domain Maxwell's equations,

$$\nabla \times \mathbf{E}(\mathbf{r}) - i\omega\mu_0\mu_\mathrm{r}(\mathbf{r})\mathbf{H}(\mathbf{r}) = 0, \tag{1a}$$
$$\nabla \times \mathbf{H}(\mathbf{r}) + i\omega\varepsilon_0\varepsilon_\mathrm{r}(\mathbf{r})\mathbf{E}(\mathbf{r}) = \mathbf{J}(\mathbf{r}), \tag{1b}$$
$$\nabla \cdot [\varepsilon_\mathrm{r}(\mathbf{r})\mathbf{E}(\mathbf{r})] = \rho(\mathbf{r})/\varepsilon_0, \tag{1c}$$
$$\nabla \cdot [\mu_\mathrm{r}(\mathbf{r})\mathbf{H}(\mathbf{r})] = 0, \tag{1d}$$

where $\mathbf{E}(\mathbf{r}) = (E_x, E_y, E_z)(\mathbf{r})$ and $\mathbf{H}(\mathbf{r}) = (H_x, H_y, H_z)(\mathbf{r})$ are the electric and magnetic fields at position $\mathbf{r} = (x, y, z)$, $\varepsilon_\mathrm{r}(\mathbf{r})$ and $\mu_\mathrm{r}(\mathbf{r})$ the scalar relative permittivity and permeability profiles that define the structure, $\mathbf{J}(\mathbf{r})$ and $\rho(\mathbf{r})$ the electric current density and charge density satisfying continuity equation $\nabla \cdot \mathbf{J}(\mathbf{r}) = i\omega\rho(\mathbf{r})$ that act as the sources, and $\varepsilon_0$ and $\mu_0$ the vacuum permittivity and permeability constants.

Consider a nonmagnetic ($\mu_\mathrm{r} = 1$) system where $\varepsilon_\mathrm{r}(\mathbf{r})$, $\mathbf{E}(\mathbf{r})$, $\mathbf{H}(\mathbf{r})$, $\mathbf{J}(\mathbf{r})$, and $\rho(\mathbf{r})$ are independent of $z$. Then, the transverse-magnetic (TM) field components $H_x$, $H_y$, and $E_z$ are decoupled from the transverse-electric (TE) components $E_x$, $E_y$, and $H_z$. Inserting the $\mathbf{H}(\mathbf{r})$ in Eq. (1a) into Eq. (1b) and taking the $z$ component, we obtain the governing equation for the TM waves,

$$\left[-\nabla_{xy}^2 - \frac{\omega^2}{c^2}\varepsilon_\mathrm{r}(x, y)\right] E_z(x, y) = i\omega\mu_0 J_z(x, y) \equiv b(x, y), \tag{2}$$

where $\nabla_{xy}^2 = \partial^2/\partial x^2 + \partial^2/\partial y^2$, and $c = 1/\sqrt{\varepsilon_0\mu_0}$ is the vacuum speed of light. We define the right-hand side $b(x, y)$ as the source profile. Once $E_z$ is solved for, the magnetic-field components of the TM wave follow from Eq. (1a) as $(H_x, H_y) = \frac{1}{i\omega\mu_0}\left(\frac{\partial E_z}{\partial y}, -\frac{\partial E_z}{\partial x}\right)$. Eqs. (1c)–(1d) are automatically satisfied. The vacuum wavelength is $\lambda = 2\pi c/\omega$.

For the scattering problems considered below, there is no physical current source, so the right-hand side $J_z$ is zero. We will put effective sources on the right-hand side of Eq. (2) to generate the incident field and to solve for the resulting scattered field, but the total field $E_z$ still satisfies Eq. (2) with $J_z = 0$; see Sec. 1 C.

Consider a structure where $\varepsilon_\mathrm{r}(x, y)$ is periodic in $y$ with periodicity $W$, namely $\varepsilon_\mathrm{r}(x, y + W) = \varepsilon_\mathrm{r}(x, y)$. Given the periodicity, any solution $E_z(x, y)$ of Eq. (2) can be written as a superposition of Bloch states with different Bloch wave numbers $k_\mathrm{B}$, with each Bloch state satisfying $E_z(x, y + W) = E_z(x, y)e^{ik_\mathrm{B}W}$. Bloch states with distinct $k_\mathrm{B}$ (up to modulo $2\pi/W$) are decoupled from each other. Therefore, we can fix $k_\mathrm{B}$ and consider only a finite transverse size within $0 \leq y \leq W$, with boundary condition $E_z(x, W) = E_z(x, 0)e^{ik_\mathrm{B}W}$ at $y = 0$ and $y = W$; this is called Bloch periodic boundary condition, which reduces to a periodic boundary when $k_\mathrm{B} = 0$.

We further consider the structure to be homogeneous on the left and right sides,

$$\varepsilon_\mathrm{r}(x, y) = \begin{cases} \varepsilon_\mathrm{L}, & x \leq 0, \\ \varepsilon_\mathrm{r}(x, y), & 0 < x < L, \\ \varepsilon_\mathrm{R}, & x \geq L. \end{cases} \tag{3}$$

Light incident from either side is scattered by the inhomogeneous structure within $0 < x < L$, which we refer to as the scattering region. The scattering matrix fully characterizes such response. To define the scattering matrix, we first define the input and output "channels" as follows.

### A. Channels in homogeneous space

Consider a homogeneous region (either $x \leq 0$ or $x \geq L$), within which $\varepsilon_\mathrm{r}(x, y) = \varepsilon_\mathrm{bg}$ is a real-valued constant (either $\varepsilon_\mathrm{L}$ or $\varepsilon_\mathrm{R}$). Given the translational symmetry in $x$, the field profile within the homogeneous region can be written as

a superposition of propagating and evanescent fields of the form

$$E_z^{(a,\pm)}(x,y) = \frac{1}{\sqrt{k_x^{(a)}}} u_a(y) e^{\pm i k_x^{(a)} x}, \quad a \in \mathbb{Z}, \tag{4}$$

which we refer to as "channels." The integer index $a$ labels the channel number, $\pm$ indicates the propagation direction, $k_x^{(a)}$ is the longitudinal wave number, the prefactor $1/\sqrt{k_x^{(a)}}$ normalizes the longitudinal flux (to be discussed in the next paragraph), and $u_a(y)$ is the transverse mode profile. The $a$-th transverse profile is

$$u_a(y) = \frac{1}{\sqrt{W}} \exp\left[ i k_y^{(a)} (y - y_0) \right], \quad 0 \le y \le W, \tag{5}$$

with the transverse wave number $k_y^{(a)} = k_{\rm B} + a\frac{2\pi}{W}$ chosen to satisfy the Bloch periodic boundary condition; note that neighboring $k_y^{(a)}$ are separated by $2\pi/W$. Here, $y_0$ is an arbitrary real constant specifying the reference position of the basis. The set $\{u_a(y)\}_a$ of transverse modes makes up a complete and orthonormal basis, with $\int_0^W dy u_a^*(y) u_b(y) = \delta_{ab}$. Inserting Eqs. (4)–(5) into Eq. (2) with no source yields the dispersion relation $(\omega/c)^2 \varepsilon_{\rm bg} = (k_x^{(a)})^2 + (k_y^{(a)})^2$. We choose the sign of $k_x^{(a)}$ as $k_x^{(a)} = \sqrt{(\omega/c)^2 \varepsilon_{\rm bg} - (k_y^{(a)})^2}$. There is an infinite number of evanescent channels where $k_x^{(a)}$ is imaginary [*i.e.*, where $|k_y^{(a)}| > (\omega/c)\sqrt{\varepsilon_{\rm bg}}$]. There are approximately $2\sqrt{\varepsilon_{\rm bg}} W/\lambda$ propagating channels where $k_x^{(a)}$ is real-valued [*i.e.*, where $|k_y^{(a)}| < (\omega/c)\sqrt{\varepsilon_{\rm bg}}$]; these are plane waves propagating at angles $\theta_a = {\rm atan}(k_y^{(a)}/k_x^{(a)})$. Therefore, $2\sqrt{\varepsilon_{\rm bg}} W/\lambda$ complex-valued coefficients are necessary to fully specify the propagating components of an arbitrary incident wavefront or outgoing wavefront; this is consistent with the Nyquist–Shannon sampling theorem [1] that when the highest spatial frequency (*i.e.*, wave number $k_y$) in a wavefront is $(\omega/c)\sqrt{\varepsilon_{\rm bg}}$, we need at least one spatial sampling point per $\lambda/(2\sqrt{\varepsilon_{\rm bg}})$ to uniquely specify such a wavefront.

The channels are flux orthogonal: given a superposition of Eq. (4) with different channel indices and propagation directions, the $x$-directional Poynting flux of the total field integrated over $y$ equals the sum of the integrated Poynting flux for components with different transverse profiles. For propagating channels, the $1/\sqrt{k_x^{(a)}}$ prefactor in Eq. (4) ensures that different propagating channels are normalized to carry the same longitudinal flux; furthermore, the integrated flux from the two counter-propagating terms have opposite signs, and the cross term does not contribute. For evanescent channels, neither $E_z^{(a,+)}$ nor $E_z^{(a,-)}$ itself carries flux, but their cross terms can carry flux.

### B. Scattering matrix

We now define the scattering matrix $\mathbf{S}$. In a scattering problem, the total field $E_z$ satisfies Eq. (2) with no source and can be written as $E_z(x,y) = E_z^{\rm in}(x,y) + E_z^{\rm sca}(x,y)$. Here, $E_z^{\rm in}$ is the incident field, and $E_z^{\rm sca}$ is the scattered field that satisfies an outgoing boundary condition at infinity (*i.e.*, when $|x| \to \infty$). To be concrete, we consider light incident from the left at angle $\theta_a$, with

$$E_z^{\rm in}(x,y) = \frac{E_0}{\sqrt{k_x^{(a,{\rm L})}}} u_a^{({\rm L})}(y) e^{i k_x^{(a,{\rm L})} x} \tag{6}$$

for some constant $E_0$. Outside of the scattering region, the total field $E_z$ can be written as a superposition of the propagating and evanescent channels consistent with the outgoing boundary condition, as

$$\frac{E_z(x,y)}{E_0} = \begin{cases} \frac{u_a^{({\rm L})}(y)}{\sqrt{k_x^{(a,{\rm L})}}} e^{i k_x^{(a,{\rm L})} x} + \sum_b r_{ba}^{({\rm L})} \frac{u_b^{({\rm L})}(y)}{\sqrt{k_x^{(b,{\rm L})}}} e^{-i k_x^{(b,{\rm L})} x} + {\sum_b}' \tilde{r}_{ba}^{({\rm L})} \frac{u_b^{({\rm L})}(y)}{\sqrt{k_x^{(b,{\rm L})}}} e^{-i k_x^{(b,{\rm L})} x}, & x \le 0, \\ \sum_b t_{ba}^{({\rm L})} \frac{u_b^{({\rm R})}(y)}{\sqrt{k_x^{(b,{\rm R})}}} e^{i k_x^{(b,{\rm R})}(x-L)} + {\sum_b}' \tilde{t}_{ba}^{({\rm L})} \frac{u_b^{({\rm R})}(y)}{\sqrt{k_x^{(b,{\rm R})}}} e^{i k_x^{(b,{\rm R})}(x-L)}, & x \ge L. \end{cases} \tag{7}$$

Summations $\sum_b$ sum over the $N_{\rm L} \approx 2\sqrt{\varepsilon_{\rm L}} W/\lambda$ and $N_{\rm R} \approx 2\sqrt{\varepsilon_{\rm R}} W/\lambda$ propagating channels on the left and right, and summations $\sum_b'$ sum over the countably infinite number of evanescent channels. Here, $r_{ba}^{({\rm L})}$ and $t_{ba}^{({\rm L})}$ are the reflection and transmission coefficients with input in channel $a$ from the left and output in channel $b$ on the left or right,

normalized by the longitudinal flux, with reference planes on $x = 0$ and $x = L$ respectively. In the absence of absorption or gain (*i.e.*, when $\varepsilon_r(x, y)$ is real-valued everywhere), flux conservation requires that $\sum_b |r_{ba}|^2 + \sum_b |t_{ba}|^2 = 1$ for any propagating channel $a$. Meanwhile, $\tilde{r}_{ba}$ and $\tilde{t}_{ba}$ characterize the evanescent response in the near field.

While it is possible to include the evanescent response in the scattering matrix [2], in most scenarios only the propagating ones are of interest. We define reflection matrix $\mathbf{r}_L$ and transmission matrix $\mathbf{t}_L$ with incidence from the left by their matrix elements $r_{ba}^{(L)}$ and $t_{ba}^{(L)}$; together they form the scattering matrix $\mathbf{S}_L = [\mathbf{r}_L; \mathbf{t}_L]$ to include output to both sides. In general, we can consider incident light from either left or right, with the full scattering matrix being

$$\mathbf{S} = \begin{bmatrix} \mathbf{r}_L & \mathbf{t}_R \\ \mathbf{t}_L & \mathbf{r}_R \end{bmatrix}. \tag{8}$$

This full scattering matrix has size $(N_L + N_R)$-by-$(N_L + N_R)$ and is unitary in the absence of absorption or gain.

### C. Fisher–Lee relation

One can introduce sources to the right-hand side of Eq. (2) to solve the scattering problem, and there are many options on the type of source to use (*e.g.*, volume sources, total-field/scattered-field sources [3, 4]). The augmented partial factorization (APF) method is more efficient when sparse source profiles are used, so we use a surface source that is nonzero only at $x' = 0$ or $x' = L$. The resulting expression for the scattering matrix is the same as the Fisher–Lee relation that was initially derived for the single-particle Schrödinger equation in quantum transport [5–7].

We first define the retarded Green's function $G(x, y; x', y')$ of the scattering medium, which is the solution of Eq. (2) with a point source at $(x', y')$,

$$\lim_{\eta \to 0^+} \left[ -\nabla_{xy}^2 - \frac{\omega^2}{c^2} \varepsilon_r(x, y) - i\eta \right] G(x, y; x', y') = \delta(x - x')\delta(y - y'). \tag{9}$$

The infinitesimal absorption $\eta$ imposes an outgoing boundary condition on $G(x, y; x', y')$.

We can then use the Green's function to express the total field $E_z$ arising from the incident field in Eq. (6), as

$$E_z(x, y) = E_z^{\text{out}}(x, y) + E_z^{\text{io}}(x, y), \tag{10a}$$

$$E_z^{\text{out}}(x, y)/E_0 = -2i\sqrt{k_x^{(a,L)}} \int_0^W dy' G(x, y; x' = 0, y') u_a^{(L)}(y'), \tag{10b}$$

$$E_z^{\text{io}}(x, y)/E_0 = \frac{1}{\sqrt{k_x^{(a,L)}}} u_a^{(L)}(y) \left( e^{ik_x^{(a,L)}x} - e^{-ik_x^{(a,L)}x} \right) H(-x), \tag{10c}$$

where $H(x) = [\text{sign}(x) + 1]/2$ is a Heaviside step function. The $E_z^{\text{out}}$ in Eq. (10b) is the solution of Eq. (2) given a surface source $b(x', y') = -2i\sqrt{k_x^{(a,L)}}\delta(x')u_a^{(L)}(y')E_0$ at $x' = 0$ and with an outgoing boundary condition. In the absence of scatterers [*i.e.*, when $\varepsilon_r(x, y) = \varepsilon_L$ everywhere], such a surface source generates an outgoing field $E_z^{\text{out}}(x, y) = \frac{E_0}{\sqrt{k_x^{(a,L)}}} u_a^{(L)}(y) e^{ik_x^{(a,L)}|x|} \equiv E_{z0}^{\text{out}}(x, y)$ that propagates away from $x = 0$ toward both sides and equals the incident field $E_z^{\text{in}}(x, y)$ in Eq. (6) on the $x \geq 0$ side; the addition of $E_z^{\text{io}}$ in Eq. (10c) subtracts the outgoing wave on the $x < 0$ side and adds $E_z^{\text{in}}(x, y)$ there, such that $E_{z0}^{\text{out}} + E_z^{\text{io}} = E_z^{\text{in}}$. In the presence of scatterers, $E_z^{\text{out}}$ additionally includes the scattered field $E_z^{\text{sca}}$ produced by such $E_z^{\text{in}}$ interacting with the scatterers, namely $E_z^{\text{out}} = E_{z0}^{\text{out}} + E_z^{\text{sca}}$. For $x \geq 0$, $E_{z}^{\text{out}} = E_z^{\text{in}}$, so $E_z^{\text{out}} = E_z^{\text{in}} + E_z^{\text{sca}} = E_z$ is the total field. More generally, $E_z^{\text{out}} = E_z - E_z^{\text{io}}$ as shown in Eq. (10a). We can validate that the $E_z$ in Eq. (10) is indeed the solution of the scattering problem because (1) $E_z^{\text{io}}$ is a solution of Eq. (2) with an opposite surface source $b'(x', y') = 2i\sqrt{k_x^{(a,L)}}\delta(x')u_a^{(L)}(y')E_0 = -b(x', y')$, so the sum $E_z = E_z^{\text{out}} + E_z^{\text{io}}$ satisfies the source-less Eq. (2) everywhere, and (2) the $E_z$ in Eq. (10) satisfies the boundary condition of the scattering problem defined by $E_z = E_z^{\text{in}} + E_z^{\text{sca}}$ with $E_z^{\text{in}}$ from Eq. (6) and an $E_z^{\text{sca}} = E_z^{\text{out}} + (E_z^{\text{io}} - E_z^{\text{in}})$ that is purely outgoing on both sides.

By equating Eq. (7) and Eq. (10), evaluating it at $x = 0$ and $x = L$ (note that only $E_z^{\text{out}}$ contributes since $E_z^{\text{io}} = 0$ at $x = 0$ and $x = L$), and projecting onto the output channels using the orthonormality of the transverse modes

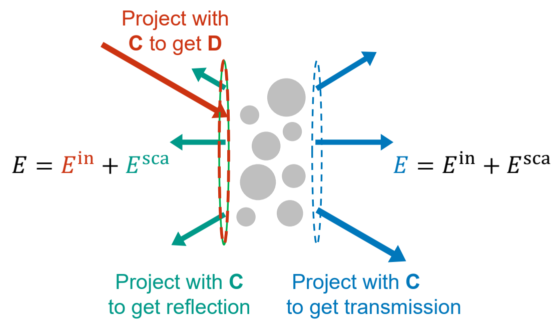


**Supplementary Fig. 1**. **Schematic illustration of $\mathbf{S} = \mathbf{CA}^{-1}\mathbf{B} - \mathbf{D}$ in Eq.** (11) **and Eq.** (24)**.** Given a surface source, the solution $\mathbf{A}^{-1}\mathbf{B}$ at $x \geq 0$ is the total field $E = E^{\rm in} + E^{\rm sca}$. Projecting such total field at $x = L$ onto the transverse modes through multiplication with matrix $\mathbf{C}$ gives the transmission matrix. Projecting the total field at $x = 0$ gives the reflection matrix (from the projection of $E^{\rm sca}$) plus $\mathbf{D}$ (from the projection of $E^{\rm in}$), so $\mathbf{D}$ is subtracted to yield the reflection matrix.

introduced in Sec. 1 A, we obtain the reflection and transmission coefficients as

$$r_{ba}^{(\rm L)} = -2i\sqrt{k_x^{(b,\rm L)}k_x^{(a,\rm L)}}\int dy \int dy' u_b^{(\rm L)*}(y)G(x=0,y;x'=0,y')u_a^{(\rm L)}(y') - \delta_{ba}, \tag{11a}$$

$$t_{ba}^{(\rm L)} = -2i\sqrt{k_x^{(b,\rm R)}k_x^{(a,\rm L)}}\int dy \int dy' u_b^{(\rm R)*}(y)G(x=L,y;x'=0,y')u_a^{(\rm L)}(y'), \tag{11b}$$

which is the Fisher–Lee relation for scalar fields [5–7].

Eq. (11) is a special case of Eq. (2) in the main text, $\mathbf{S} = \mathbf{CA}^{-1}\mathbf{B} - \mathbf{D}$, and can be understood intuitively: the integrations become matrix-matrix multiplications when discretized; matrix $\mathbf{A}^{-1}$ is the retarded Green's function; for each incident angle $\theta_a$, $b_a(x',y') = -2i\sqrt{k_x^{(a,\rm L)}}\delta(x')u_a^{(\rm L)}(y')$ is the input source, corresponding to one column of matrix $\mathbf{B}$; the output projection matrix $\mathbf{C}$ corresponds to $\sqrt{k_x^{(b,\rm L)}}\delta(x)u_b^{(\rm L)*}(y)$ and $\sqrt{k_x^{(b,\rm R)*}}\delta(x-L)u_b^{(\rm R)}(y)$ for reflection and transmission coefficients into different outgoing angles $\theta_b$; matrix $\mathbf{D}$ is the $\delta_{ba}$ in Eq. (11a), which is the projection of $E_z^{\rm in}$ on the incident side since reflection is the projection of $E_z^{\rm sca} = E_z - E_z^{\rm in}$. This is schematically illustrated in **Supplementary Fig. 1**, which is a visualization of the matrix operations in Fig. 1c of the main text. Explicit definition of the matrices $\mathbf{A}$, $\mathbf{B}$, $\mathbf{C}$, $\mathbf{D}$ are given in the next section.

## 2. FINITE-DIFFERENCE DISCRETIZATION

We need to discretize the system in order to compute its scattering matrix numerically. Here we consider finite-difference discretization on the Yee grid [8]. On the Yee grid, the first derivatives are approximated with center difference with second-order accuracy, and the grid points of different field components are staggered in space. For 2D TM waves, we put the discretized $E_z$ component at

$$E_z(x = x_n, y = y_m) \to E_{z(n,m)} \tag{12}$$

where

$$x_n \equiv \left(n - \frac{1}{2}\right)\Delta x, \quad y_m \equiv \left(m - \frac{1}{2}\right)\Delta x \tag{13}$$

with $(n,m)$ being integer indices and $\Delta x$ the discretization grid size. The $H_x$ component is located at $(x_n, y_{m+1/2})$, and the $H_y$ component is located at $(x_{n+1/2}, y_m)$. This gives the discretized second derivative of $E_z$ in $x$ as

$$\left.\frac{\partial^2}{\partial x^2}E_z(x,y)\right|_{x=x_n,y=y_m} \to \frac{E_{z(n+1,m)} - 2E_{z(n,m)} + E_{z(n-1,m)}}{\Delta x^2}, \tag{14}$$

and similarly for $\partial^2 E_z/\partial y^2$. We discretize the relative permittivity profile $\varepsilon_{\rm r}(x,y)$ through subpixel smoothing [9]; for TM waves, this corresponds simply to averaging $\varepsilon_{\rm r}(x,y)$ within the $\Delta x^2$ area of each cell centered at $(x_n, y_m)$, as

$$\varepsilon_{n,m} = \frac{1}{\Delta x^2}\int_{(n-1)\Delta x}^{n\Delta x} dx \int_{(m-1)\Delta x}^{m\Delta x} dy\, \varepsilon_{\rm r}(x,y). \tag{15}$$

Note that different from the notation of Ref. [8], in Eq. (13) we introduced the half-pixel offset so that the first pixel $(n,m)=(1,1)$ of $E_z$ will have its lower corner at $(x,y)=(0,0)$; this is more convenient when we only deal with $E_z$. Then, the differential operator $-\nabla_{xy}^2 - (\omega/c)^2\varepsilon_{\rm r}(x,y)$ of Eq. (2) is discretized into $A_{(n,m),(n',m')}/\Delta x^2$ with

$$A_{(n,m),(n',m')} = -(\delta_{n+1,n'}+\delta_{n-1,n'})\delta_{m,m'} + (4-\beta^2\varepsilon_{n,m})\delta_{n,n'}\delta_{m,m'} - (\delta_{m+1,m'}+\delta_{m-1,m'})\delta_{n,n'} \tag{16}$$

away from the boundaries, with $\beta \equiv (\omega/c)\Delta x$. This is the finite-difference frequency-domain (FDFD) formulation.

In practice, the system size is made finite with $N'$ pixels, and a single index is used to go through both indices $(n,m)$. With that single index, $E_{z(n,m)}$ becomes an $N'\times 1$ column vector $\mathbf{E}_z$, the source profile $b_{(n,m)}$ also becomes an $N'\times 1$ column vector $\mathbf{b}$, and $A_{(n,m),(n',m')}$ becomes an $N'\times N'$ square matrix $\mathbf{A}$. Then, Eq. (2) becomes a system of linear equations expressed through matrix-vector multiplication,

$$\mathbf{A}\mathbf{E}_z = \mathbf{b}. \tag{17}$$

Now we consider the two-sided geometry in Sec. 1. We restrict the integer index $m$ to $1 \le m \le n_y$ with $n_y \equiv W/\Delta x$ being the transverse number of grid points (discretizing the region $0 \le y \le W$); $\Delta x$ can be chosen such that $n_y$ is an integer. The Bloch periodic boundary condition $E_{z,(n,n_y+1)} = E_{z,(n,1)}e^{ik_{\rm B}n_y\Delta x}$ and $E_{z,(n,0)} = E_{z,(n,n_y)}e^{-ik_{\rm B}n_y\Delta x}$ manifests in the boundary elements of matrix $\mathbf{A}$. The system size in $x$ is infinite, but we must truncate it to a finite size with an effective open boundary with no reflection at the interface of truncation; we do so with the perfectly matched layer (PML) [10], which attenuates the outgoing waves with minimal reflection. In this way, the outgoing boundary condition is also built into matrix $\mathbf{A}$.

The retarded Green's function $G(x,y;x',y')$ defined in Eq. (9) is the inverse of the differential operator. When discretized, it becomes a matrix and is simply given by the matrix inverse

$$\mathbf{G} = \mathbf{A}^{-1}. \tag{18}$$

The outgoing property of $\mathbf{G}$ follows from the PML in matrix $\mathbf{A}$.

Under subpixel smoothing, Eq. (3) becomes

$$\varepsilon_{n,m} = \begin{cases} \varepsilon_{\rm L}, & n \le 0, \quad 1 \le m \le n_y, \\ \varepsilon_{n,m}, & 1 \le n \le n_x, \quad 1 \le m \le n_y, \\ \varepsilon_{\rm R}, & n \ge n_x+1, \quad 1 \le m \le n_y, \end{cases} \tag{19}$$

where $n_x \equiv \lceil L/\Delta x \rceil$ is the number of grid points for the scattering region. The rest follows the same steps as in Sec. 1, which we summarize below.

### A. Channels in discrete homogeneous space

Given the discrete translational symmetry in $n$ when $n \le 0$ and $n \ge n_x+1$, Eq. (4) still holds in the form of

$$E_{z(n,m)}^{(a,\pm)} = \frac{1}{\sqrt{\nu_a}} u_{ma} \exp\left[\pm i k_x^{(a)} \Delta x \left(n - \frac{1}{2}\right)\right], \tag{20}$$

with the longitudinal-flux normalization factor $\nu_a$ to be determined. The transverse modes of Eq. (5) become

$$u_{ma} = \frac{1}{\sqrt{n_y}} \exp\left[i k_y^{(a)} \Delta x\, (m - m_0)\right], \tag{21}$$

which make up matrix $\mathbf{u}$, with $y_0 = (m_0 - 1/2)\Delta x$. The transverse wave number is still $k_y^{(a)} = k_{\rm B} + a\frac{2\pi}{n_y\Delta x}$, but channel $a$ is now equivalent to channel $a+n_y$ due to aliasing, so there are only $n_y$ distinct channels in total (propagating ones plus evanescent ones) instead of an infinite number of them. Completeness and orthonormality of the transverse

modes means that the $n_y \times n_y$ matrix $\mathbf{u}$ is unitary when all $n_y$ channels are included. Inserting Eqs. (20)–(21) into Eq. (17) with $b = 0$ yields the finite-difference dispersion relation

$$\varepsilon_{\rm bg} \left(\frac{\omega}{c}\right)^2 \Delta x^2 = 4 \sin^2 \left(\frac{k_x^{(a)} \Delta x}{2}\right) + 4 \sin^2 \left(\frac{k_y^{(a)} \Delta x}{2}\right). \tag{22}$$

Flux orthogonality continues to hold in the discretized system. To preserve flux conservation (*i.e.*, Poynting's theorem), the Poynting vector in the discrete system should be defined using the product of an $\mathbf{E}$ field component and an $\mathbf{H}$ field component at $\Delta x/2$ away [11]. For 2D TM fields, this means that the $x$-directional flux is proportional to $\mathrm{Im}\left[E^*_{z(n,m)} E_{z(n+1,m)}\right]$. For each channel in Eq. (20), such flux is proportional to $\frac{\pm 1}{|\nu_a|} \sin\left(k_x^{(a)} \Delta x\right)$ for propagating channels (where $k_x^{(a)}$ is real-valued), zero for evanescent channels (where $k_x^{(a)}$ is imaginary or complex-valued). Therefore, we choose the flux normalization factor in Eq. (20) to be

$$\nu_a = \sin\left(k_x^{(a)} \Delta x\right) \tag{23}$$

so that all propagating channels carry equal longitudinal flux.

### B. Finite-difference Fisher–Lee relation

The definition of the scattering matrix is the same as the continuous case in Eq. (7), simply with $x$ and $y$ replaced through Eq. (13).

The derivation of the scattering matrix in terms of the Green's function and surface sources (namely, the Fisher–Lee relation) is the same as the continuous case except for one difference: the sources and the projections were placed at $x = 0$ and $x = L$ in the continuous case, but the corresponding spatial indices $n = (x/\Delta x) + (1/2)$ would lie on $n = 1/2$ and $n = (L/\Delta x) + (1/2)$, which are generally not integer points. Therefore, for the discretized system, we put the sources and the projections at integer indices $n = 0$ and $n = n_x + 1$ (recall that $n_x \equiv \lceil L/\Delta x \rceil$). This gives the discrete version of Eq. (11) as

$$r_{ba}^{(\rm L)} = \left[-2i\sqrt{\nu_b^{(\rm L)} \nu_a^{(\rm L)}} \sum_m \sum_{m'} u_{mb}^{(\rm L)*} G_{(n=0,m),(n'=0,m')} u_{m'a}^{(\rm L)} - \delta_{ba}\right] \delta_b^{(\rm L)} \delta_a^{(\rm L)}, \tag{24a}$$

$$t_{ba}^{(\rm L)} = \left[-2i\sqrt{\nu_b^{(\rm R)} \nu_a^{(\rm L)}} \sum_m \sum_{m'} u_{mb}^{(\rm R)*} G_{(n=n_x+1,m),(n'=0,m')} u_{m'a}^{(\rm L)}\right] \delta_b^{(\rm R)} \delta_a^{(\rm L)}. \tag{24b}$$

Here, $\delta_b^{(\rm L/R)} = \exp\left[-ik_x^{(b,\rm L/R)} \Delta x \delta n^{(\rm L/R)}\right]$ and $\delta_a^{(\rm L/R)} = \exp\left[-ik_x^{(a,\rm L/R)} \Delta x \delta n^{(\rm L/R)}\right]$ are phase factors that compensate for the half-pixel shift of the detection plane and the source plane respectively, with $\delta n^{(\rm L)} = 1/2$, $\delta n^{(\rm R)} = 1/2 + \lceil L/\Delta x \rceil - (L/\Delta x)$; these factors can be ignored if the precise location of the reference plane is not of interest.

Based on Eq. (18), Eq. (24), and considering inputs from both sides as in Eq. (8), we can write the full scattering matrix $\mathbf{S}$ as

$$\mathbf{S} = \mathbf{C}\mathbf{A}^{-1}\mathbf{B} - \mathbf{D}, \tag{25}$$

which is unitary in the absence of absorption or gain if all propagating channels are included. A schematic illustration of Eqs. (24)–(25) is given in **Supplementary Fig. 1** and Fig. 1c of the main text.

Specifically, matrix $\mathbf{A}$ is $\Delta x^2$ times the discrete version of the differential operator $-\nabla^2_{xy} - (\omega/c)^2 \varepsilon_{\rm r}(x, y)$ in Eq. (2), given by Eq. (16), PML, and the boundary conditions. The input matrix $\mathbf{B}$ is

$$\mathbf{B} = \begin{bmatrix} \mathbf{0} & \mathbf{0} \\ \mathbf{B}_{\rm L} & \mathbf{0} \\ \mathbf{0} & \mathbf{0} \\ \mathbf{0} & \mathbf{B}_{\rm R} \\ \mathbf{0} & \mathbf{0} \end{bmatrix}. \tag{26}$$

The top (bottom) block row of zeros correspond to indices $n < 0$ ($n > n_x + 1$) with PML and homogeneous space on the left (right); they are shown in green in Fig. 1c–d of the main text. The second (fourth) block row corresponds to

index $n = 0$ ($n = n_x + 1$), which is the left (right) surface where input sources are placed; they are shown in red in Fig. 1c–d of the main text. The third block row corresponds to the scattering region with indices $1 \le n \le n_x$, shown in blue in Fig. 1c–d of the main text. Matrices $\mathbf{B}_\mathrm{L}$ and $\mathbf{B}_\mathrm{R}$ have sizes $n_y \times M_\mathrm{L}$ and $n_y \times M_\mathrm{R}$; they are line sources on the surface, given by

$$\mathbf{B}_\mathrm{L} = -2i\mathbf{u}_\mathrm{L}\sqrt{\nu_\mathrm{L}}\delta^{(\mathrm{L})}, \quad \mathbf{B}_\mathrm{R} = -2i\mathbf{u}_\mathrm{R}\sqrt{\nu_\mathrm{R}}\delta^{(\mathrm{R})}, \tag{27}$$

where the $n_y \times M_\mathrm{L}$ matrix $\mathbf{u}_\mathrm{L}$ is defined in Eq. (21), matrices $\sqrt{\nu_\mathrm{L}} = \mathrm{diag}\left(\left\{\sqrt{\nu_a^{(\mathrm{L})}}\right\}_a\right)$ and $\delta^{(\mathrm{L})} = \mathrm{diag}\left(\left\{\delta_a^{(\mathrm{L})}\right\}_a\right)$ are $M_\mathrm{L} \times M_\mathrm{L}$ diagonal matrices for flux normalization and phase shift respectively, and similarly with $\mathbf{B}_\mathrm{R}$. Similarly, output matrix $\mathbf{C}$ performs the projection onto the output channels,

$$\mathbf{C} = \begin{bmatrix} \mathbf{0} & \mathbf{C}_\mathrm{L} & \mathbf{0} & \mathbf{0} & \mathbf{0} \\ \mathbf{0} & \mathbf{0} & \mathbf{0} & \mathbf{C}_\mathrm{R} & \mathbf{0} \end{bmatrix}, \tag{28}$$

with

$$\mathbf{C}_\mathrm{L} = \delta^{(\mathrm{L})}\sqrt{\nu_\mathrm{L}}\mathbf{u}_\mathrm{L}^\dagger, \quad \mathbf{C}_\mathrm{R} = \delta^{(\mathrm{R})}\sqrt{\nu_\mathrm{R}}\mathbf{u}_\mathrm{R}^\dagger, \tag{29}$$

where $^\dagger$ stands for conjugate transpose. Note that the list of the $M' = M'_\mathrm{L} + M'_\mathrm{R}$ output channels do not have to be the same as the list of the $M = M_\mathrm{L} + M_\mathrm{R}$ input channels, but for simplicity we do not introduce a separate notation. Matrix $\mathbf{D}$ here is the $\delta_{ba}$ in the Fisher–Lee relation and is the projection of the incident field, with elements that equal $\delta_b^{(\mathrm{L/R})}\delta_a^{(\mathrm{L/R})}$ when the input channel is the same as the output channel, 0 otherwise. All of these are illustrated in **Supplementary Fig. 1** and Fig. 1c of the main text, as in Eq. (11).

For the numerical computation and implementation, it is faster and simpler to take the prefactor $-2i$ in Eq. (27) and the $\sqrt{\nu_\mathrm{L/R}}\delta^{(\mathrm{L/R})}$ prefactors in Eq. (27) and Eq. (29) out of $\mathbf{B}_\mathrm{L/R}$ and $\mathbf{C}_\mathrm{L/R}$, and multiply such prefactors after $\mathbf{CA}^{-1}\mathbf{B}$ is computed. This means we can use

$$\mathbf{B}_\mathrm{L} = \mathbf{u}_\mathrm{L}, \quad \mathbf{B}_\mathrm{R} = \mathbf{u}_\mathrm{R}, \quad \mathbf{C}_\mathrm{L} = \mathbf{u}_\mathrm{L}^\dagger, \quad \mathbf{C}_\mathrm{R} = \mathbf{u}_\mathrm{R}^\dagger, \tag{30}$$

instead for the numerical computations. We omit $\mathbf{D}$ from matrix $\mathbf{K}$ [as in Eq. (31) below] and subtract $\mathbf{D}$ from $\mathbf{CA}^{-1}\mathbf{B}$ after the prefactors are put back.

While the details are system dependent, the concepts above are general, and scattering matrices can always be written in the form of Eq. (25) regardless of the discretization scheme, the geometry, the type of sources, and the type of outputs of interest. For all of these different scenarios, the baseline contribution can always be written as $\mathbf{D} = \mathbf{CA}_0^{-1}\mathbf{B} - \mathbf{S}_0$, where $\mathbf{A}_0$ is the Maxwell operator of a reference system (*e.g.*, a homogeneous one) for which the scattering matrix $\mathbf{S}_0$ is known. In the specific case above, $\mathbf{CA}_0^{-1}\mathbf{B} = \mathbf{I} + \mathbf{S}_0$ where the identity matrix $\mathbf{I}$ comes from projection of $E_z^\mathrm{in}$ on the incident side (assuming the full scattering matrix and ignoring the phase-shift factors to simplify notation); in general, this $\mathbf{D}$ provides the proper baseline such that $\mathbf{S}$ reduces to $\mathbf{S}_0$ when $\mathbf{A}$ becomes $\mathbf{A}_0$.

## 3. UTILIZING SYMMETRY IN AUGMENTED PARTIAL FACTORIZATION (APF)

In APF, a partial factorization is performed on the augmented sparse matrix

$$\mathbf{K} \equiv \begin{bmatrix} \mathbf{A} & \mathbf{B} \\ \mathbf{C} & \mathbf{0} \end{bmatrix}. \tag{31}$$

For most linear solvers such as the MUMPS package [12] we use, the computing time and memory usage of such partial factorization can be reduced when matrix $\mathbf{K}$ is symmetric. Thanks to reciprocity, the bulk of matrix $\mathbf{A}$ as in Eq. (16) is symmetric; periodic boundary condition and the use of uniaxial PML in 2D does not break such symmetry. Therefore, matrix $\mathbf{K}$ will be symmetric if we can make $\mathbf{C} = \mathbf{B}^\mathrm{T}$, or equivalently $\mathbf{C}_\mathrm{L} = \mathbf{B}_\mathrm{L}^\mathrm{T}$ and $\mathbf{C}_\mathrm{R} = \mathbf{B}_\mathrm{R}^\mathrm{T}$.

From Eq. (30), we see that when the list of input channels equals the list of output channels, we would have the desired $\mathbf{C}_\mathrm{L} = \mathbf{B}_\mathrm{L}^\mathrm{T}$ if the transverse mode profiles were real-valued. The transverse mode profiles in Eq. (21) are not real-valued, but we can see that taking the complex conjugate of the profile is equivalent to flipping the sign of $k_y^{(a)}$; with a periodic boundary condition in $y$ (where $k_\mathrm{B} = 0$), this corresponds to flipping the sign of the channel index $a$. Therefore, we can achieve $\mathbf{C}_\mathrm{L} = \mathbf{B}_\mathrm{L}^\mathrm{T}$ simply by making the list of output channels having the opposite channel index as the list of input channels. When the full scattering matrix or reflection matrix is computed, all we need to do is to

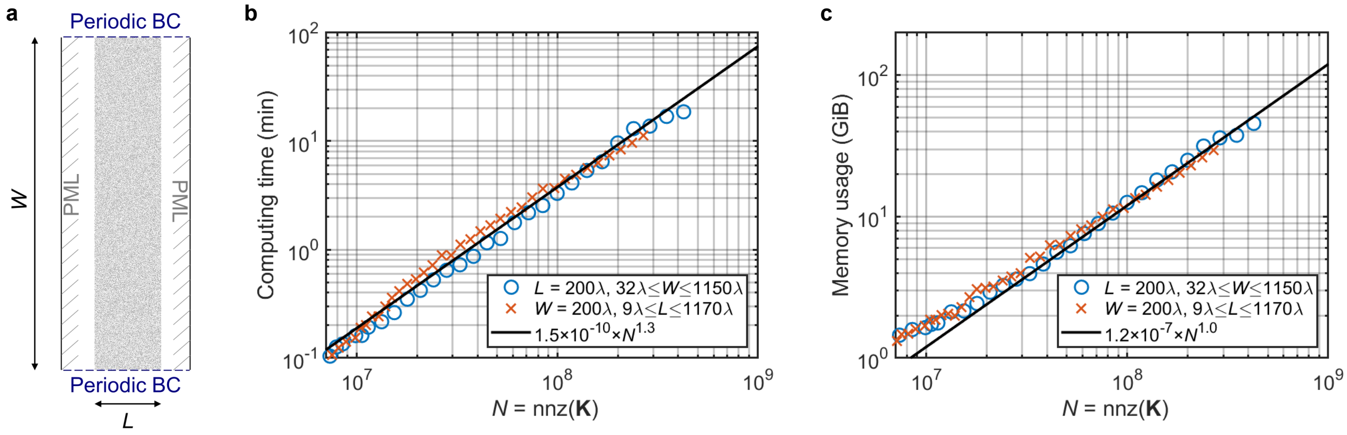


**Supplementary Fig. 2**. **Computing time and memory usage of APF**. **a**, Schematic of the disordered system considered, with thickness $L$ and width $W$. Other parameters are the same as in Fig. 2 of the main text. **b–c**, Computing time (**b**) and memory usage (**c**) versus the number $N$ of nonzero elements in matrix $\mathbf{K}$. Symbols are from simulations; black lines are fitting curves.

choose a particular ordering of the channels (which can be reversed after $\mathbf{CA}^{-1}\mathbf{B}$ is computed). When only a subset of the scattering matrix is needed, we can further pad input and/or output channels to achieve $\mathbf{C}_\mathrm{L} = \mathbf{B}_\mathrm{L}^\mathrm{T}$ to make matrix $\mathbf{K}$ symmetric. Note this is applicable to any structure and does not require any symmetry in $\varepsilon_\mathrm{r}(x, y)$.

## 4. COMPUTING TIME AND MEMORY SCALING OF APF

We implement APF under finite-difference discretization for 2D TM fields as described above, and here we map out its computing time and memory usage scaling as a function of the system size. We consider the same disordered system as in Fig. 2 of the main text but with different system sizes: (1) fixing thickness at $L = 200\lambda$ while varying width $W$ from 32 $\lambda$ to 1150 $\lambda$, and (2) fixing $W = 200\lambda$ while varying $L$ from 9 $\lambda$ to 1170 $\lambda$. The computing time and memory usage are obtained with serial computations on Intel Xeon Gold 6130 nodes. For each system size, the full scattering matrix (with $M' = M \approx 4W/\lambda$) is computed 10 times; the average computing time and the maximal recorded memory usage among the 10 computations is used. A constant 0.57 GiB memory used by MATLAB R2020b is subtracted from the memory usage.

The computing time and memory usage are well characterized by the number $N$ of nonzero elements in the sparse matrix $\mathbf{K}$ [denoted by $\mathrm{nnz}(\mathbf{K})$], as shown in **Supplementary Fig. 2b–c**. An $\mathcal{O}(N^{1.3})$ curve and an $\mathcal{O}(N)$ curve closely describe the computing time and the memory usage for all of these systems.

In the systems benchmarked here, $\mathrm{nnz}(\mathbf{A})/\mathrm{nnz}(\mathbf{B})$ ranges between 1 and 100, and $\mathrm{nnz}(\mathbf{A})/(M'M)$ ranges between 3.5 and 460. Therefore, $\mathrm{nnz}(\mathbf{K}) \approx \mathrm{nnz}(\mathbf{A})$ is proportional to the number of pixels in the discretization, and the APF computing time and memory usage are independent of the number $M$ of input channels.

## 5. APF WITH COMPRESSED INPUT/OUTPUT MATRICES (APF-C)

When $\mathrm{nnz}(\mathbf{B})$ or $\mathrm{nnz}(\mathbf{C})$ exceeds $\mathrm{nnz}(\mathbf{A})$, the computing time and memory usage of APF can grow with the number of inputs $M$ or the number of outputs $M'$, which is not desirable. But there is a simple solution: we can "compress" matrices $\mathbf{B}$ and $\mathbf{C}$ to reduce their number of nonzero elements, perform the partial factorization, and then "decompress." Conceptually, this is similar to other forms of data compression. We call this APF-c where c stands for compression.

In this section, we consider a simple compression scheme based on the Fourier transform, which is more than sufficient for the metasurface examples considered in this paper; note when the accuracy is sufficient and when $\mathrm{nnz}(\mathbf{B})$ and $\mathrm{nnz}(\mathbf{C})$ goes below $\mathrm{nnz}(\mathbf{A})$, further compression is no longer necessary. More advanced compression strategies [13] may be used if there is such need.

**Supplementary Fig. 3a** illustrates the expression of the scattering matrix $\mathbf{S}$ in Eq. (25), highlighting the nonzero blocks $\mathbf{B}_\mathrm{L}$, $\mathbf{B}_\mathrm{R}$ of the input matrix $\mathbf{B}$ and the nonzero blocks $\mathbf{C}_\mathrm{L}$, $\mathbf{C}_\mathrm{R}$ of the output matrix $\mathbf{C}$. These nonzero blocks are placed on the front ($x = 0$) and back ($x = L$) surfaces of the scattering region, as indicated in red in Fig. 1d of

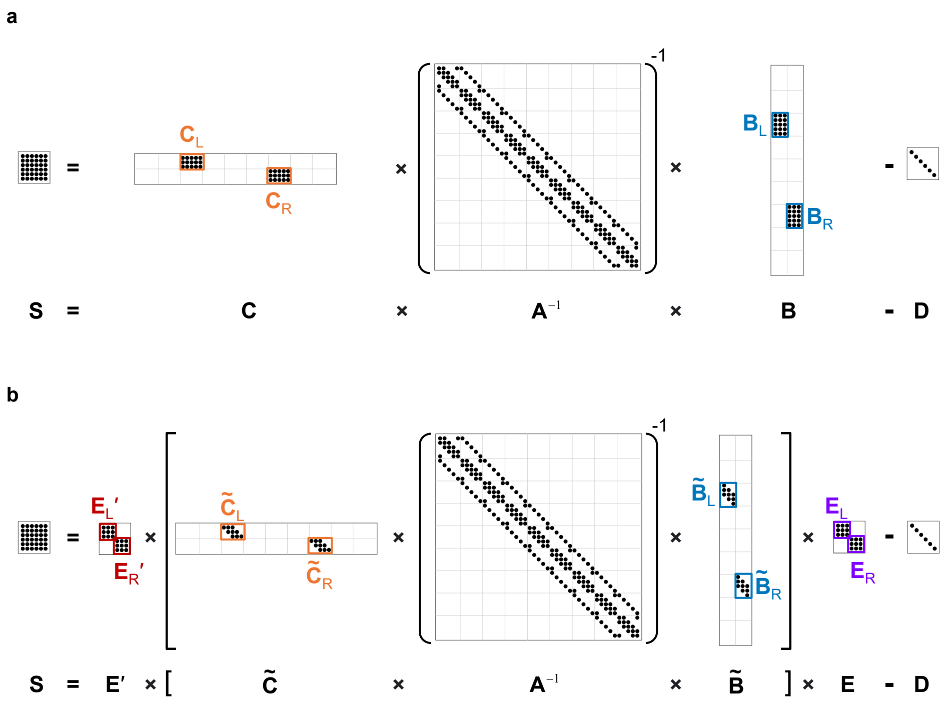


**Supplementary Fig. 3**. **Concept of APF with compression (APF-c)**. **a**, Schematic illustration of Eq. (25) that highlights the dense blocks $\mathbf{B}_{\rm L}$, $\mathbf{B}_{\rm R}$ and $\mathbf{C}_{\rm L}$, $\mathbf{C}_{\rm R}$ of the input and output matrices. **b**, With APF-c, the input and output matrices are transformed such that $\tilde{\mathbf{B}}_{\rm L}$, $\tilde{\mathbf{B}}_{\rm R}$, $\tilde{\mathbf{C}}_{\rm L}$, $\tilde{\mathbf{C}}_{\rm R}$ are spatially localized (see **Supplementary Fig. 4**) and can be truncated with minimal or no loss of accuracy. The transformations are reversed after $\tilde{\mathbf{C}}\mathbf{A}^{-1}\tilde{\mathbf{B}}$ is computed.

the main text. Even though matrices $\mathbf{B}$ and $\mathbf{C}$ are nonzero only on these two surfaces, these nonzero blocks are dense and can still contain many elements when the system is wide. Each column of $\mathbf{B}_{\rm L}$ is the cross section of a plane wave given in Eq. (21), which covers the full range of $y$ as shown in **Supplementary Fig. 4a**.

While a single plane wave is extended in space, a superposition of plane waves can form a sharp focus, with the focus location determined by the relative phases of the constituting plane waves [14, 15]. And such a conversion between angular basis (plane waves) and spatial basis (focused waves) is invertible. With this idea in mind, we will take a discrete Fourier transform of the dense block $\mathbf{B}_{\rm L}$ along its channel index $a$, and similarly with $\mathbf{B}_{\rm R}$, $\mathbf{C}_{\rm L}$, and $\mathbf{C}_{\rm R}$. For concreteness, below we consider having $M_{\rm L}$ input channels from the left with consecutive channel indices $a = -\frac{M_{\rm L}-1}{2}, \cdots, 0, \cdots, \frac{M_{\rm L}-1}{2}$, under periodic boundary condition with $m_0 = 0$. We define an $M_{\rm L}\times M_{\rm L}$ discrete Fourier transform (DFT) matrix $\mathbf{F}_{M_{\rm L}}$ by its elements,

$$(\mathbf{F}_{M_{\rm L}})_{ba} = \frac{1}{\sqrt{M_{\rm L}}} e^{-i\frac{2\pi}{M_{\rm L}}ba}. \tag{32}$$

The inverse DFT matrix is then $\mathbf{F}_{M_{\rm L}}^{-1} = \mathbf{F}_{M_{\rm L}}^{\dagger}$. Note that the indices $b$ and $a$ here are centered around zero; the DFT matrix is commonly defined with indices starting at zero instead, which is equivalent to the definition here after an index shift.

When taking a superposition of plane waves to form a focus, the weight of the constituting plane waves can determine how fast the intensity decays away from the focal spot. Therefore, we also introduce a diagonal matrix $\mathbf{Q}_{M_{\rm L}}$, defined as

$$\mathbf{Q}_{M_{\rm L}} = \mathrm{diag}\left(\left\{q_a^{M_{\rm L}}\right\}\right), \tag{33}$$

where $\left\{q_a^{M_{\rm L}}\right\}_a$ are nonzero real numbers (the weights).

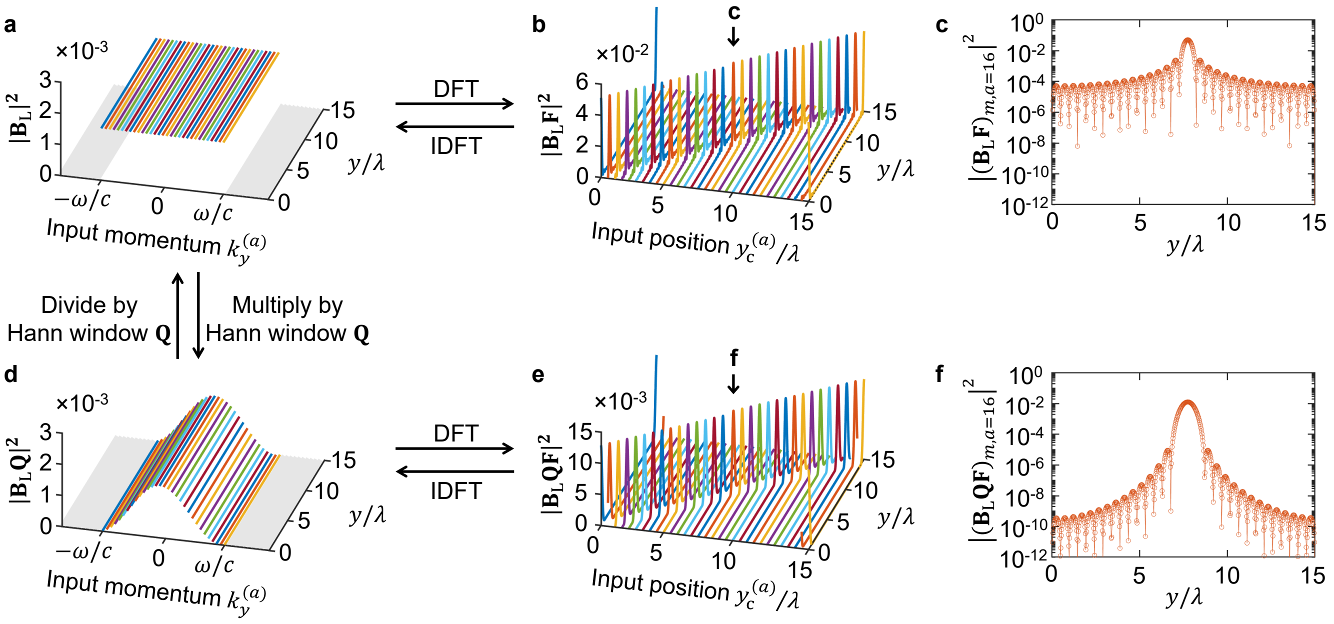


**Supplementary Fig. 4**. **Transformations on the input matrix in APF-c.** **a**, Original input matrix $\mathbf{B}_{\mathrm{L}}$; each column spans the full width of the system. **b**, Input matrix after a discrete Fourier transform, $\tilde{\mathbf{B}}_{\mathrm{L}} = \mathbf{B}_{\mathrm{L}}\mathbf{F}$; each column is now spatially localized. **c**, The central column of $\tilde{\mathbf{B}}_{\mathrm{L}}$ in **b**, in log scale. **d**, Input matrix $\mathbf{B}_{\mathrm{L}}\mathbf{Q}$ weighted by the Hann window function. **e**, Input matrix after Hann window and discrete Fourier transform, $\tilde{\mathbf{B}}_{\mathrm{L}} = \mathbf{B}_{\mathrm{L}}\mathbf{Q}\mathbf{F}$. **f**, The central column of $\tilde{\mathbf{B}}_{\mathrm{L}}$ in **e**, in log scale; the Hann window makes the tails decay faster. The system considered here has width $W = 15\lambda$ discretized with $\Delta x = \lambda/40$. The subscript on the number of channels is dropped to simplify notation.

Then, matrix $\mathbf{B}_{\mathrm{L}}$ can be written as

$$\mathbf{B}_{\mathrm{L}} = \mathbf{u}_{\mathrm{L}} = \underbrace{\left(\mathbf{u}_{\mathrm{L}}\mathbf{Q}_{M_{\mathrm{L}}}\mathbf{F}_{M_{\mathrm{L}}}\right)}_{\equiv \tilde{\mathbf{B}}_{\mathrm{L}}}\underbrace{\left(\mathbf{F}_{M_{\mathrm{L}}}^{-1}\mathbf{Q}_{M_{\mathrm{L}}}^{-1}\right)}_{\equiv \mathbf{E}_{\mathrm{L}}}. \tag{34}$$

Similarly,

$$\mathbf{B}_{\mathrm{R}} = \mathbf{u}_{\mathrm{R}} = \left(\mathbf{u}_{\mathrm{R}}\mathbf{Q}_{M_{\mathrm{R}}}\mathbf{F}_{M_{\mathrm{R}}}\right)\left(\mathbf{F}_{M_{\mathrm{R}}}^{-1}\mathbf{Q}_{M_{\mathrm{R}}}^{-1}\right) \equiv \tilde{\mathbf{B}}_{\mathrm{R}}\mathbf{E}_{\mathrm{R}}, \tag{35a}$$

$$\mathbf{C}_{\mathrm{L}} = \mathbf{u}_{\mathrm{L}}^{\dagger} = \left(\mathbf{Q}_{M_{\mathrm{L}}}^{-1}\mathbf{F}_{M_{\mathrm{L}}}\right)\left(\mathbf{F}_{M_{\mathrm{L}}}^{-1}\mathbf{Q}_{M_{\mathrm{L}}}\mathbf{u}_{\mathrm{L}}^{\dagger}\right) \equiv \mathbf{E}'_{\mathrm{L}}\tilde{\mathbf{C}}_{\mathrm{L}}, \tag{35b}$$

$$\mathbf{C}_{\mathrm{R}} = \mathbf{u}_{\mathrm{R}}^{\dagger} = \left(\mathbf{Q}_{M_{\mathrm{R}}}^{-1}\mathbf{F}_{M_{\mathrm{R}}}\right)\left(\mathbf{F}_{M_{\mathrm{R}}}^{-1}\mathbf{Q}_{M_{\mathrm{R}}}\mathbf{u}_{\mathrm{R}}^{\dagger}\right) \equiv \mathbf{E}'_{\mathrm{R}}\tilde{\mathbf{C}}_{\mathrm{R}}, \tag{35c}$$

The transformed input and output matrices $\tilde{\mathbf{B}}$ and $\tilde{\mathbf{C}}$ can be defined just like Eq. (26) and Eq. (28). Instead of computing $\mathbf{C}\mathbf{A}^{-1}\mathbf{B}$, we can compute $\tilde{\mathbf{C}}\mathbf{A}^{-1}\tilde{\mathbf{B}}$ with APF using the transformed input/output matrices, after which we undo the transformations using $\mathbf{E}_{\mathrm{L}}$, $\mathbf{E}_{\mathrm{R}}$, $\mathbf{E}'_{\mathrm{L}}$, $\mathbf{E}'_{\mathrm{R}}$. The procedure is illustrated in **Supplementary Fig. 3b**. So far, we have not introduced compression yet; the transformed matrices take up the same sizes as the original matrices, and the transformations can be reversed with no approximation.

While the columns of the original matrix $\mathbf{B}_{\mathrm{L}}$ are spatially extended, those of the transformed matrix $\tilde{\mathbf{B}}_{\mathrm{L}}$ can be made spatially localized. Without the weights (*i.e.* $\mathbf{Q}_{M_{\mathrm{L}}} = \mathbf{I}$), the matrix elements of $\tilde{\mathbf{B}}_{\mathrm{L}}$ can be analytically derived from Eq. (21) and Eq. (32) as

$$(\mathbf{u}_{\mathrm{L}}\mathbf{F}_{M_{\mathrm{L}}})_{ma} = \sum\nolimits_{b=-(M_L-1)/2}^{(M_L-1)/2}(\mathbf{u}_{\mathrm{L}})_{mb}(\mathbf{F}_{M_{\mathrm{L}}})_{ba} = \begin{cases} \dfrac{(-1)^a}{\sqrt{n_y M_{\mathrm{L}}}}\dfrac{\sin\left(\pi m M_{\mathrm{L}}/n_y\right)}{\sin\left(\frac{\pi m}{n_y} - \frac{\pi a}{M_{\mathrm{L}}}\right)}, & \text{when } \dfrac{m}{n_y} - \dfrac{a}{M_{\mathrm{L}}} \notin \mathbb{Z}, \\ \dfrac{M_{\mathrm{L}}}{\sqrt{n_y M_{\mathrm{L}}}}, & \text{otherwise}, \end{cases} \tag{36}$$

which is the discrete form of the sinc function coming from Fourier transforming a rectangular window (*i.e.*, $|k_y| < \omega/c$)

in momentum space. The $a$-th column of $\tilde{\mathbf{B}}_\mathrm{L}$ in Eq. (36) is peaked around a center point $y_\mathrm{c}^{(a)} \equiv (m_\mathrm{c}^{(a)} - 0.5)\Delta x$ with $m_\mathrm{c}^{(a)} = \mathrm{mod}(n_y a/M_\mathrm{L}, n_y)$, near which its envelope decays as $1/\left|y - y_\mathrm{c}^{(a)}\right|$ since $\left|\sin\left(\frac{\pi m}{n_y} - \frac{\pi a}{M_\mathrm{L}}\right)\right| \approx \frac{\pi}{n_y}|m - m_\mathrm{c}^{(a)}|$ in the denominator. **Supplementary Fig. 4a–b** plot the columns of $\mathbf{B}_\mathrm{L}$ and the columns of the transformed matrix $\tilde{\mathbf{B}}_\mathrm{L}$ with no weight.

Given the spatial localization of $\tilde{\mathbf{B}}_\mathrm{L}$, we can specify a truncation window width $w_\mathrm{t}$ and set the matrix elements of $\tilde{\mathbf{B}}_\mathrm{L}$ with $|y - y_\mathrm{c}^{(a)}| > w_\mathrm{t}/2$ to zero to make matrix $\tilde{\mathbf{B}}_\mathrm{L}$ sparse—this is the compression step. Such truncation significantly reduces $\mathrm{nnz}(\tilde{\mathbf{B}}_\mathrm{L})$, by a factor of $w_\mathrm{t}/W$. There is no need to build the full matrix $\mathbf{u}_\mathrm{L}$; we only need to build the elements of $\tilde{\mathbf{B}}_\mathrm{L}$ within the truncation window $w_\mathrm{t}$ using Eq. (36). There is no need to build the DFT matrix $\mathbf{F}_{M_\mathrm{L}}$ either, since the transformations can be reversed efficiently with fast Fourier transforms [16].

The truncation does introduce small errors since the elements dropped were not exactly zero before. **Supplementary Fig. 4c** plots one column of $\tilde{\mathbf{B}}_\mathrm{L}$ in log scale, which makes the nonzero nature of the $1/\left|y - y_\mathrm{c}^{(a)}\right|$ tail more visible. To reduce the compression error, we can increase the window size $w_\mathrm{t}$ and/or make the columns of $\tilde{\mathbf{B}}_\mathrm{L}$ decay faster. The relatively slow $1/|y - y_\mathrm{c}^{(a)}|$ decay in real space comes from the sharp edges of $|k_y| < \omega/c$ in momentum space. So, we can make $\tilde{\mathbf{B}}_\mathrm{L}$ more localized using weights that smoothen the sharp edges. Here, we use the Hann window function [17]:

$$q_a^M = \frac{1}{2}\left[1 + \cos\left(\frac{2\pi a}{M}\right)\right]. \tag{37}$$

As Eq. (37) is a superposition of exponential functions, the matrix elements of $\tilde{\mathbf{B}}_\mathrm{L} = \mathbf{u}_\mathrm{L}\mathbf{Q}_{M_\mathrm{L}}\mathbf{F}_{M_\mathrm{L}}$ can be readily derived as

$$(\mathbf{u}_\mathrm{L}\mathbf{Q}_{M_\mathrm{L}}\mathbf{F}_{M_\mathrm{L}})_{ma} = \frac{1}{2}(\mathbf{u}_\mathrm{L}\mathbf{F}_{M_\mathrm{L}})_{ma} + \frac{1}{4}(\mathbf{u}_\mathrm{L}\mathbf{F}_{M_\mathrm{L}})_{m,a-1} + \frac{1}{4}(\mathbf{u}_\mathrm{L}\mathbf{F}_{M_\mathrm{L}})_{m,a+1}, \tag{38}$$

where each term is given by Eq. (36). **Supplementary Fig. 4d–e** illustrate this process. With the Hann window, the columns of $\tilde{\mathbf{B}}_\mathrm{L}$ now decays significantly faster as $1/|y - y_\mathrm{c}^{(a)}|^3$, shown in **Supplementary Fig. 4f**.

In **Supplementary Fig. 10** of Sec. 15, we provide detailed comparison of the compression error for the mm-wide metasurface examples considered in the main text. There, we can see that a direct use of the Hann window actually increases the compression error despite the faster decay of the tails. This is because when we undo the weights through $\mathbf{Q}^{-1}$ in Eqs. (34)–(35c), the compression error at large angles are significantly amplified. Since the error amplification only occurs at large angles, it can be mitigated by padding $M_\mathrm{pad}$ extra channels at larger $|k_y|$; the padded channels will then suffer significant error amplification from $\mathbf{Q}^{-1}$, but they will be discarded. The original channels then avoid the error amplification from $\mathbf{Q}^{-1}$. These padded channels do not need to be propagating channels; evanescent ones work equally well since only the transverse profile is involved. Such padding has the additional benefit of increasing $M_\mathrm{L}$, which reduces the width of the $\sin(\pi m M_\mathrm{L}/n_y)$ term in the numerator of Eq. (36), making $\tilde{\mathbf{B}}_\mathrm{L}$ more localized with less compression error when truncated. (In the limit when the padded $M_\mathrm{L}$ reaches $n_y$, each column of $\tilde{\mathbf{B}}_\mathrm{L}$ will be zero everywhere except at the pixel of $m = m_\mathrm{c}^{(a)}$, so the compression error completely vanishes even when the truncation window $w_\mathrm{t}$ is a single-pixel wide.) Indeed, **Supplementary Fig. 10** shows that when additional channels are padded, the compression error with Hann window drops sharply to become orders of magnitude lower than the error without Hann window. Note, however, that if the padding raises the number of elements of $\mathbf{S}$ substantially above $\mathrm{nnz}(\mathbf{A})$, the computation time and memory usage will also increase (see Sec. 9).

Lastly, we note that $\tilde{\mathbf{B}}_\mathrm{L}$ in Eq. (36) and Eq. (38) is already real-valued, so $\tilde{\mathbf{C}}_\mathrm{L} = \tilde{\mathbf{B}}_\mathrm{L}^\mathrm{T}$ whenever $M_\mathrm{L} = M_\mathrm{L}'$. The channel-index flipping described in Sec. 3 to make matrix $\mathbf{K}$ symmetric is no longer necessary.

## 6. APF PSEUDOCODE

The pseudocodes of APF and APF-c are shown below, which is also the structure of our implementation made open-source at [18]. One can specify an arbitrary system contained in input argument `syst` (including the permittivity profile, wavelength, discretization grid size, boundary conditions, and PML parameters), arbitrary lists of source profiles given by matrix $\mathbf{B}$, and arbitrary output projections given by matrix $\mathbf{C}$. The algorithm returns the scattering matrix $\mathbf{S}$.

**Algorithm 1** APF

**Input:** syst, $\mathbf{B}$, $\mathbf{C}$, $\mathbf{D}$ ▷ syst specifies the system; $\mathbf{B}$, $\mathbf{C}$ specify the inputs and outputs; $\mathbf{D} = \mathbf{C}\mathbf{A}_0^{-1}\mathbf{B} - \mathbf{S}_0$.
**Output:** $\mathbf{S}$
- $\mathbf{A} \leftarrow$ syst ▷ Build the sparse matrix $\mathbf{A}$ for the Maxwell operator.
- $\mathbf{K} = [\mathbf{A}, \mathbf{B}; \mathbf{C}, \mathbf{0}]$ ▷ Build the augmented matrix $\mathbf{K}$.
- $\mathbf{H} = \mathbf{K}/\mathbf{A}$ ▷ Compute the Schur complement $\mathbf{K}/\mathbf{A} = -\mathbf{C}\mathbf{A}^{-1}\mathbf{B}$.
- $\mathbf{S} = -\mathbf{H} - \mathbf{D}$ ▷ Scattering matrix $\mathbf{S} = \mathbf{C}\mathbf{A}^{-1}\mathbf{B} - \mathbf{D}$.
- **return** $\mathbf{S}$

**Algorithm 2** APF-c

**Input:** syst, $\mathbf{B}$, $\mathbf{C}$, $\mathbf{D}$ ▷ syst specifies the system; $\mathbf{B}$, $\mathbf{C}$ specify the inputs and outputs; $\mathbf{D} = \mathbf{C}\mathbf{A}_0^{-1}\mathbf{B} - \mathbf{S}_0$.
**Output:** $\mathbf{S}$
- $\mathbf{A} \leftarrow$ syst ▷ Build the sparse matrix $\mathbf{A}$ for the Maxwell operator.
- $\tilde{\mathbf{B}}, \mathbf{E}, \tilde{\mathbf{C}}, \mathbf{E}' \leftarrow \mathbf{B}, \mathbf{C}$ ▷ Compress the matrices $\mathbf{B}$ and $\mathbf{C}$.
- $\tilde{\mathbf{K}} = [\mathbf{A}, \tilde{\mathbf{B}}; \tilde{\mathbf{C}}, \mathbf{0}]$ ▷ Build the augmented matrix $\tilde{\mathbf{K}}$.
- $\tilde{\mathbf{H}} = \tilde{\mathbf{K}}/\mathbf{A}$ ▷ Compute the Schur complement $\tilde{\mathbf{K}}/\mathbf{A} = -\tilde{\mathbf{C}}\mathbf{A}^{-1}\tilde{\mathbf{B}}$.
- $\mathbf{S} = -\mathbf{E}'\tilde{\mathbf{H}}\mathbf{E} - \mathbf{D}$ ▷ Decompress with the matrices $\mathbf{E}$ and $\mathbf{E}'$.
- **return** $\mathbf{S}$

## 7. SYSTEM-SIZE SCALING FOR DISORDERED MEDIA SIMULATIONS

**Supplementary Fig. 5** shows how the computing time and memory usage scale with the system size using different methods, for the disordered system in Fig. 2 of the main text with the aspect ratio $W/L = 5$ fixed while the overall system size ($W$ and $L$) varies. The full scattering matrix (with $M' = M \approx 4W/\lambda$) is computed. Here, $\mathrm{nnz}(\mathbf{A})/\mathrm{nnz}(\mathbf{B}) \approx 4$ and $\mathrm{nnz}(\mathbf{A})/(M'M) \approx 15$ stay roughly unchanged as the system size varies. Some methods require more computing resources than we have access to, so their data points (open symbols) are extrapolated from smaller number of input angles $M$ and/or smaller systems. We note that among all of these methods, APF exhibits the best scaling both in terms of computing time and in terms of memory usage, and is many orders of magnitude faster for large systems.

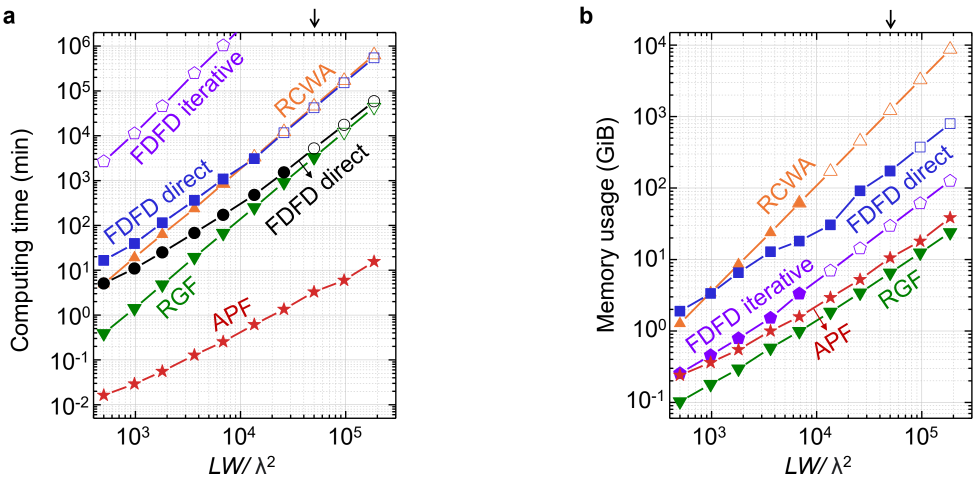


**Supplementary Fig. 5**. **Scaling of computing time and memory usage using different methods**. Computing time (**a**) and memory usage (**b**) versus the system size $LW/\lambda^2$. The two "FDFD direct" curves correspond to an unmodified version of MaxwellFDFD (blue curve) and one modified to have the LU factors reused for different inputs (black curve). The arrows on top indicate the system considered in Fig. 2 of the main text.

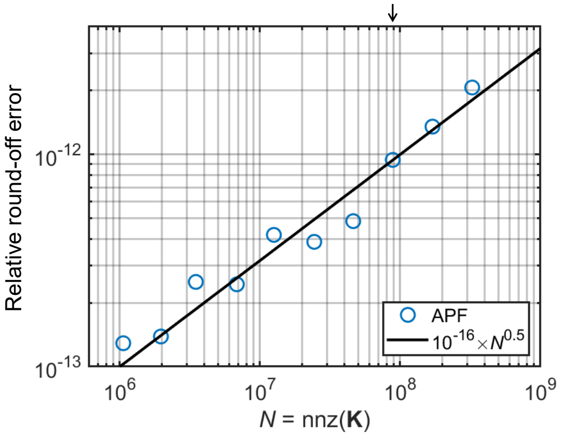


**Supplementary Fig. 6**. **Round-off error of APF**. Circles show the relative difference between scattering matrices computed using APF and those computed using direct method with iterative refinement that iterates until machine precision is reached. Black line is a fitting curve. The black arrow on top indicates the system considered in Fig. 2 of the main text.

## 8. ROUND-OFF ERROR OF APF

While APF is in theory exact aside from discretization error (and compression error if APF-c is used), numerical round off is also present, and such round-off errors often grow with the system size. Therefore, it is necessary to check if the round-off error of APF is acceptably small. To characterize the round-off error, we compare results from APF to those obtained from a standard direct solver but with additional iterative refinement [19] steps that iterate until the entire solution (with all input channels) reaches machine-precision accuracy. Double precision is used through out.

From that, we evaluate the relative $\ell^2$-norm error, $\|\mathbf{S}_{\rm APF} - \mathbf{S}_0\|_2 / \|\mathbf{S}_0\|_2$, for the systems in Sec. 7, where $\mathbf{S_0}$ is the scattering matrix (reshaped into a vector) computed with iterative refinement, and $\mathbf{S}_{\rm APF}$ is that from APF. The difference between the two is the round-off error of APF. The relative round-off error with respect to $N = {\rm nnz}(\mathbf{K})$ scales as $\mathcal{O}(N^{0.5})$ (**Supplementary Fig. 6**); it is only $10^{-12}$ for the system considered in Fig. 2 of the main text where $N \approx 10^8$, and is estimated to be only $10^{-10}$ even for an extremely large system with a trillion matrix elements. We therefore conclude that the round-off error of APF is negligible even for the largest system one would possibly simulate.

## 9. APF FOR VERY LARGE SCATTERING MATRICES

In addition to ${\rm nnz}(\mathbf{A}) > {\rm nnz}(\mathbf{B})$, the $M$-independence of the APF computing time and memory usage also requires ${\rm nnz}(\mathbf{A}) > M'M$ where $M'M$ is the number of elements in the generalized scattering matrix $\mathbf{S}$. In this section, we explore what happens when matrix $\mathbf{S}$ becomes so large that the latter condition is no longer met.

Since matrix $\mathbf{S}$ contains $M'M$ elements, computing these elements and storing them must take at least $\mathcal{O}(M'M)$ time and memory even in the best-case scenario, regardless of the method used. So, in the limit of $M$ becoming very large, the optimal scaling of computing time and memory usage would be $\mathcal{O}(M'M)$. Intuitively, we expect such $\mathcal{O}(M'M)$ scaling to take over in APF when matrix $\mathbf{S}$ contains more elements than ${\rm nnz}(\mathbf{A})$. To see what the actual scaling is and where the transition occurs, here we consider the disordered system in Fig. 2 of the main text, but place the input sources and output projections at $M$ points in the interior of the scattering medium instead. This allows more inputs and outputs than those of propagating fields (where $M \approx 4W/\lambda$ is the upper limit). The generalized scattering matrix in this case is the retarded Green's function $G(\mathbf{r}, \mathbf{r}')$ of Eq. (9) evaluated at such pairs of points. We use the same detection points as the source points (so, $M' = M$), with these points evenly spaced inside the medium; the spacing is decreased progressively to raise $M$ up to $M = 44,000$. **Supplementary Fig. 7** shows the computing time and memory usage of APF and MaxwellFDFD. The red dotted lines show fittings to a second-order polynomial in $M$, and the black dashed lines indicate $M_0 = \sqrt{{\rm nnz}(\mathbf{A})} \approx 7,600$, above which $M'M > {\rm nnz}(\mathbf{A})$. We indeed observe the APF computing time and memory usage to remain constant up to $M = M_0$. When $M > M_0$, they both exhibit $\mathcal{O}(M'M) = \mathcal{O}(M^2)$ scaling, which is the best-case one can expect at large $M$.

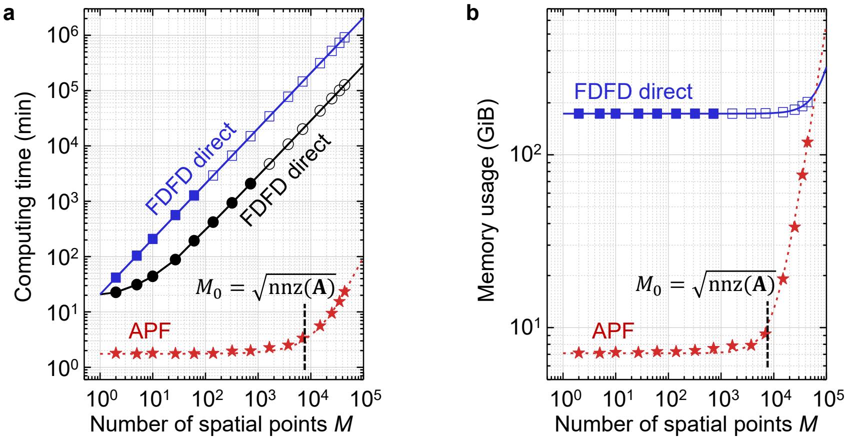


**Supplementary Fig. 7**. **Timing and memory usage for computing the Green's function.** Computing time (**a**) and memory usage (**b**) versus the number of spatial points when computing the retarded Green's function $G(\mathbf{r}, \mathbf{r}')$ at $M$ source points $\mathbf{r}'$ and $M' = M$ detection points $\mathbf{r}$ inside the disordered system in Fig. 2 of the main text. Red dotted lines show fittings to a second-order polynomial in $M$, and black dashed lines indicate $M_0 = \sqrt{\mathrm{nnz}(\mathbf{A})} \approx 7,600$, above which the computed Green's function has more data points than $\mathrm{nnz}(\mathbf{A})$. The two "FDFD direct" curves correspond to an unmodified version of MaxwellFDFD (blue squares) and one modified to have the LU factors reused for different inputs (black circles), with open symbols being estimations from smaller $M$.

## 10. METALENS DESIGN AND SIMULATION

We start by describing how the hyperbolic and quadratic metalenses in Figs. 4–5 of the main text are designed. The metalens operates at wavelength $\lambda = 532$ nm and consists of 4,178 unit cells. Each unit cell (*i.e*, a meta-atom) has a titanium dioxide ($TiO_2$) ridge (refractive index $n = 2.43$) sitting on a silica substrate ($n = 1.46$) in air ($n = 1$), as shown in **Supplementary Fig. 8a**. The ridge height in $x$ is fixed at $L = 600$ nm, and the width $\Lambda$ of an unit cell is fixed at 239.4 nm. We consider ridge widths between 45 nm and 200 nm. We use a grid size of $\Delta x = \lambda/40$ for the finite-difference discretization, which ensures that the transmission phase shifts are accurate to within 0.1 radian (see Sec. 16). We then map out the phase and amplitude of the zeroth-order (*i.e.*, $a = b = 0$) transmission coefficient of the unit cell with Bloch periodic boundary condition in $y$, for different ridge widths and different incident angles, as shown in **Supplementary Fig. 8b–c**. From these results, we pick eight ridge widths as indicated by the green arrows in **Supplementary Fig. 8b–c** and summarized in **Supplementary Table 1**, which provide eight equally-spaced transmission phase shifts covering 0 to $2\pi$ at normal incidence.

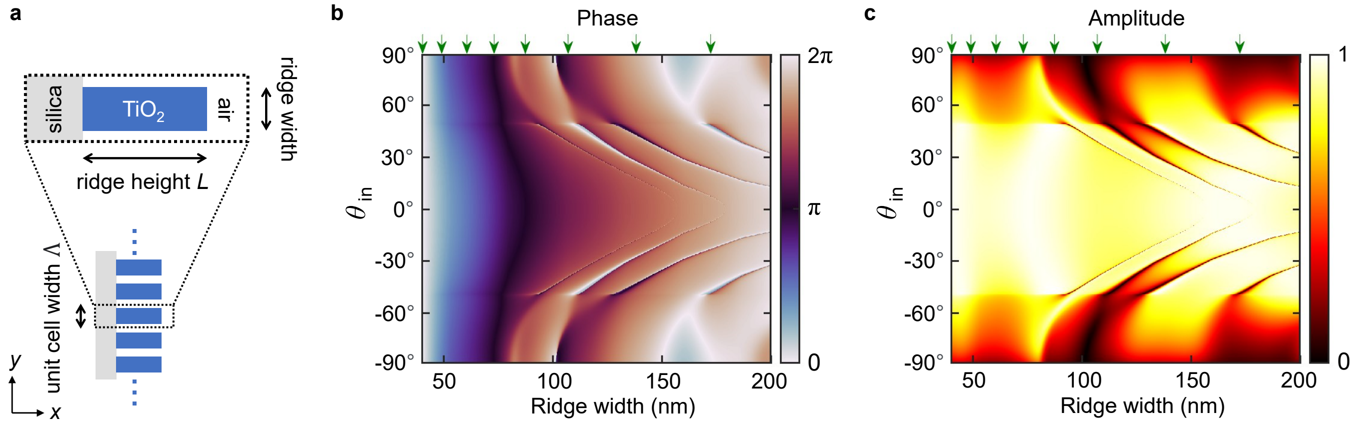


**Supplementary Fig. 8**. **Unit cells of the metasurface**. **a**, Schematic structure of a periodic array of unit cells considered here. One unit cell is simulated with Bloch periodic boundary condition in $y$. **b-c**, Phase (**b**) and amplitude (**c**) maps of the zeroth-order transmission coefficient for different ridge widths and incident angles. The green arrows on top indicate the ridge widths used for the design.

| Relative phase shift | 0 | $\pi/4$ | $\pi/2$ | $3\pi/4$ | $\pi$ | $5\pi/4$ | $3\pi/2$ | $7\pi/4$ |
|---|---|---|---|---|---|---|---|---|
| Ridge width (nm) | 40.0 | 49.1 | 60.7 | 73.1 | 87.4 | 107.1 | 138.2 | 172.3 |

**Supplementary Table 1**. **Ridge widths used for the metalens design**. Eight ridge widths and the corresponding relative transmission phase shifts at normal incidence.

For a hyperbolic metalens, the transmission phase shift at normal incidence should be space-dependent with a hyperbolic profile [20]

$$\Phi_{\text{hyperbolic}}(y) = -\frac{2\pi}{\lambda}\sqrt{f^2 + y^2}, \tag{39}$$

where $f$ is the focal length. The coordinate $y$ here is zero at the center of the metalens. A hyperbolic metalens can achieve diffraction-limited focusing at normal incidence but comes with off-axis aberrations at oblique incidence [21]. For a quadratic metalens, the transmission phase shift should follow a quadratic profile [22]

$$\Phi_{\text{quadratic}}(y) = -\frac{2\pi}{\lambda}\frac{y^2}{2f}. \tag{40}$$

We construct the metalenses from the eight unit cells in **Supplementary Table 1**, with the ridge width of each unit cell chosen based on the desired normal-incidence transmission phase shift at the center of that unit cell. The metalenses consists of 4,178 unit cells, with an overall width of $W = 1000.2$ μm. For the hyperbolic metalens, we use a focal length of $f = 300$ μm (corresponding to numerical aperture NA = 0.86). For quadratic metalenses, there is a maximal effective numerical aperture of $\text{NA}_{\text{eff}} = n_{\text{t}}/\sqrt{2}$ where $n_{\text{t}}$ is the refractive index of the medium on the transmitted side [23], so we use a larger focal length of $f = 500$ μm (corresponding to NA = 0.71).

Such design, although standard, is quite simplistic as it only considers normal incidence and assumes that the unit-cell simulations (which are carried out for fully periodic systems) are sufficient for capturing the response of the aperiodic metalens. The purpose of this design here is not to realize a high-performance metalens, but simply to provide a test system to benchmark the APF method.

After building $\varepsilon_{\text{r}}(x, y)$ of the mm-wide metalens, we carry out full-wave simulations with APF-c to compute its transmission matrix. The simulation domain is schematically illustrated in Fig. 4a of the main text, with PML on all four sides to describe a fully open system; also see the Methods section of the main text. The pre-compression inputs are surface sources in the silica substrate immediately behind the $TiO_2$ ridges, which generate incident plane waves truncated with a rectangular window over the width $W$ of the metalens; this models the effect of having an aperture that blocks incident light beyond width $W_{\text{in}} = W$. We use the set of propagating input channels for a periodic boundary in $y$ with width $W_{\text{in}}$, and restrict to the $2W_{\text{in}}/\lambda = 3{,}761$ incident angles with $|\theta_{\text{in}}^{\text{substrate}}| \leq \text{asin}(1/n_{\text{substrate}}) = 43°$ (namely, $|\theta_{\text{in}}| \leq 90°$ in air); as described in Sec. 1 A, this is the minimal number of channels we need to specify wavefronts incident from air prior to entering the substrate. For the output projections, we take the total field $E_z(x = L, y)$ immediately after the $TiO_2$ ridges across a width $W_{\text{out}} = W + 40\lambda$ in $y$ that is sufficiently large to contain all of the transmitted light, and project it onto the $2W_{\text{out}}/\lambda = 3{,}841$ propagating plane waves in air with $|\theta_{\text{out}}| \leq 90°$; this is the minimal number of output projections we need to specify the propagating components of the transmitted light. The input and output matrices are compressed for the computations, following the steps in Sec. 5.

## 11. SYSTEM-SIZE SCALING FOR METASURFACE SIMULATIONS WITH RCWA AND RGF

As RCWA and RGF work with dense matrices, they do not scale well with the system size, and simulating the mm-wide metasurface requires more computing resources than we have access to. Therefore, we extrapolate from smaller systems. We fix the thickness of the metasurface, and run RCWA and RGF simulations with increasing metasurface width $W$; the computing time and memory usage are shown as blue circles in **Supplementary Fig. 9**. For both methods, the computing time scales as $\mathcal{O}(W^3)$, which is the time scaling to invert and to multiply size-$\mathcal{O}(W)$ dense matrices; we fit the data with $\mathcal{O}(W^3)$ curves (green dashed lines) and extrapolate to a mm-wide metasurface (red circle). Similarly, the memory usage scales as $\mathcal{O}(W^2)$, which we use for extrapolation.

As described in the Methods section of the main text, the RCWA and RGF simulations here adopt a periodic boundary condition in $y$ with width $W$ (not $W_{\text{out}}$), without free space and without PML. So, the numbers here are a lower bound of the would-be numbers when an open boundary in $y$ is implemented.

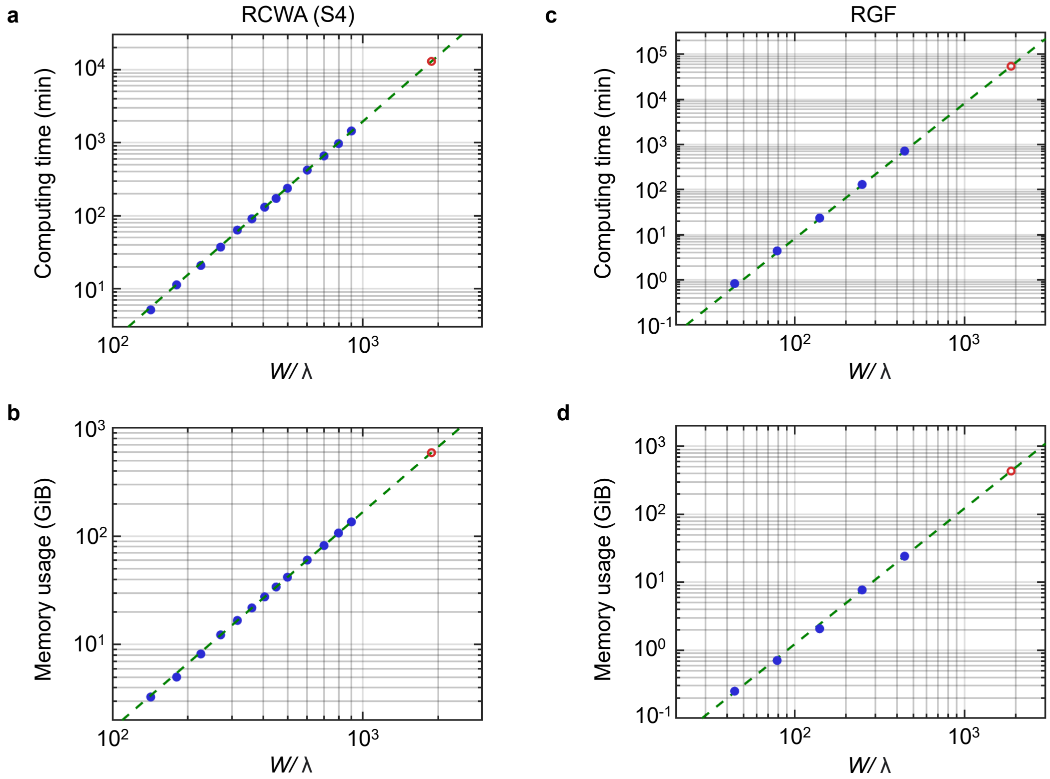


**Supplementary Fig. 9**. **System-size scaling for metasurface simulations with RCWA and RGF**. **a-b**, Computing time and memory usage of RCWA to simulate metasurfaces with varying widths $W$. **c-d**, Corresponding results with RGF. Blue circles are from simulations, green dashed lines are $\mathcal{O}(W^3)$ and $\mathcal{O}(W^2)$ fitting curves, and red circles are the points we extrapolate to estimate the computing time and memory usage for simulating a mm-wide metasurface, as shown in Fig. 4 of the main text.

## 12. ANGULAR SPECTRUM PROPAGATION

We use angular spectrum propagation (ASP) to obtain field profile in the free space after the metalens. We express $E_z\left(x,y\right)$ in terms of its Fourier components $\tilde{E}_z\left(x,k_y\right)$ as

$$E_z\left(x,y\right) = \frac{1}{\sqrt{2\pi}} \int_{-\infty}^{\infty} dk_y\, \tilde{E}_z\left(x,k_y\right) e^{ik_y y}. \tag{41}$$

Plugging Eq. (41) into the source-free Eq. (2) with $x \geq L$ [where $\varepsilon_{\mathrm{r}}(x,y) = 1$] gives $\frac{\partial^2}{\partial x^2}\tilde{E}_z\left(x,k_y\right) = -k_x^2$ with $k_x = \sqrt{(\omega/c)^2 - k_y^2}$. As there is no light incident from the right, light must propagate or decay to the right, so

$$\tilde{E}_z\left(x \geq L, k_y\right) = \tilde{E}_z\left(x = L, k_y\right) e^{ik_x(x-L)}. \tag{42}$$

Therefore, given the total field $E_z\left(x = L, y\right)$ immediately after the metasurface, we can take its Fourier transform to obtain $\tilde{E}_z\left(x = L, k_y\right)$, propagate it forward with Eq. (42), and obtain the field profile anywhere in the free space after the metalens with Eq. (41). This method is called "angular spectrum propagation" (ASP) [24].

The evanescent components (for which $|k_y| > \omega/c$) decay exponentially. Since we are not interested in the near-field close to the metasurface, we can ignore the evanescent components, and replace the $\int_{-\infty}^{\infty} dk_y$ integration range with $\int_{-\omega/c}^{\omega/c} dk_y$. Therefore, to perform ASP, we only need the propagating Fourier components of $E_z\left(x = L, y\right)$, which are precisely what's contained in the transmission matrix we computed with APF-c.

There is one subtlety. In practice, the continuous integration over $k_y$ must be approximated with a discrete summation. The transmission matrix elements have the transverse wave number $k_y$ of the transmitted light evenly spaced by $2\pi/W_{\mathrm{out}}$ as described below Eq. (5). If we perform ASP and the approximated integration directly with

such $2\pi/W_{\rm out}$ momentum spacing, it will introduce an artificial periodic boundary with periodicity $W_{\rm out}$; light that reaches the boundary will wrap around and reenter from the other side instead of leaving the domain of interest. Therefore, we need a transverse momentum spacing finer than $2\pi/W_{\rm out}$. To achieve so, we first evaluate $E_z\,(x = L, y)$ using Eq. (7), restricting the summation over $b$ to the propagating components contained in the computed transmission matrix. Then, we evaluate its Fourier components

$$\tilde{E}_z\,(x = L, k_y) = \frac{1}{\sqrt{2\pi}} \int_{-\infty}^{\infty} dy\, E_z\,(x = L, y)\, e^{-ik_y y} \tag{43}$$

on a finer grid of transverse momentum $k_y$ spaced by $2\pi/W_{\rm ASP}$. Here, we use $W_{\rm ASP} \approx 2W$, which is sufficient for eliminating the periodic wrapping artifacts within the domain of interest ($x \lesssim f$, $|y| < W/2$).

The integration range $\int_{-\infty}^{\infty} dy$ in Eq. (43) can be truncated to the width $W_{\rm out}$ where we perform the output projections, since the field beyond such window is negligibly small. A high resolution of $\Delta x = \lambda/40$ is not necessary for this spatial integration since the refractive index is lower ($n = 1$ in air) and since only the propagating components (which vary slowly in $y$) are of interest, so we use a coarser resolution of $\Delta x' = \lambda/8$ for the integration in Eq. (43). The evaluations of Eq. (7), Eq. (43), and then Eq. (41) can all be done efficiently with fast Fourier transforms [16].

ASP is an exact method; when the continuous integrals are not replaced by discrete summations, ASP is mathematically equivalent to the Rayleigh–Sommerfeld diffraction integral [25] used in Ref. [26] where it was referred to as the equivalent-current formulation.

## 13. METALENS TRANSMISSION EFFICIENCY AND STREHL RATIO

The transmission efficiency and the Strehl ratio are important metrics for assessing the performance of a metalens. Here we evaluate the incident-angle dependence of these quantities using the transmission matrix computed with APF-c. We define the transmission efficiency as the total transmitted flux in $x$ direction divided by the total incident flux in $x$ direction. Since our definition of the transmission matrix [as in Eq. (7)] is already flux-normalized, the transmission efficiency of a truncated incident plane wave with angle $\theta_{\rm in}^{(a)}$ is simply

$$T_a = \sum_b |t_{ba}|^2. \tag{44}$$

The Strehl ratio is defined as the actual intensity at the focal spot $|E_z(x = L + f, y = y_{\rm f})|^2$ divided by the would-be intensity $|E_z^{(\rm ideal)}(\mathbf{r}_{\rm f})|^2$ at the focus if perfect diffraction-limited focusing were achieved with the given transmission efficiency. Since the focus location $y_{\rm f}$ depends on the incident angle and is not clearly defined at large angles where aberrations are significant, we evaluate $|E_z(x = L + f, y = y_{\rm f})|^2$ as $\max_y |E_z(x = L + f, y)|^2$. With the transmission efficiency adjusted, the Strehl ratio is therefore

$$\mathrm{SR}(\theta_{\rm in}^{(a)}) = \frac{\max\limits_y \left|E_z^{(a)}(x = L + f, y)\right|^2 / T_a}{|E_z^{(\rm ideal)}(\mathbf{r}_{\rm f})|^2 / T_{\rm ideal}}, \tag{45}$$

where the field profile $E_z^{(a)}(x = L + f, y)$ with incident angle $\theta_{\rm in}^{(a)}$ is computed with angular spectrum propagation as described in Sec. 12. To compute $|E_z^{(\rm ideal)}(\mathbf{r}_{\rm f})|^2/T_{\rm ideal}$, we let the ideal field profile immediately after the metalens be $E_z^{(\rm ideal)}(x = L, y) = e^{i\Phi_{\rm hyperbolic}(y)}$ as in Eq. 39; its associated transmission efficiency $T_{\rm ideal}$ and field at the focus $E_z^{(\rm ideal)}(\mathbf{r}_{\rm f}) = E_z^{(\rm ideal)}(x = L + f, y = 0)$ are then evaluated with the same procedure as above.

## 14. LOCALLY PERIODIC APPROXIMATIONS

Here we consider the locally periodic approximation (LPA), which is commonly used when full-wave simulations of the entire metasurface take too much computing resources. As the field $E_z^{(a)}(x = L, y)$ immediately after the metasurface is the only input for the transmission efficiency and angular spectrum propagation, here we use LPA to approximate $E_z^{(a)}(x = L, y)$. We consider two LPA formalisms, referred to as LPA I and LPA II here. In both formalisms, the approach is to divide the metasurface into individual unit cells and assume that the response of each unit cell can be described by the unit-cell simulations in Sec. 10 and **Supplementary Fig. 8** performed for an individual unit cell under Bloch periodic boundary condition. The unit-cell simulations have incident fields

$E_z^{\text{in},p}(x,y) = \frac{E_0}{\sqrt{\Lambda k_x^{(a,\text{L})}}} \exp\left[ik_x^{(a,\text{L})}x + ik_y^{(a,\text{L})}(y-y_p)\right]$ where $y_p = (p-1)\Lambda$ is the starting position of the $p$-th unit cell, while for the full-metasurface simulation we want the incident field to be $E_z^{\text{in}}(x,y) = \frac{E_0}{\sqrt{Wk_x^{(a,\text{L})}}} \exp\left[ik_x^{(a,\text{L})}x + ik_y^{(a,\text{L})}y\right]$, so a prefactor of $\sqrt{\Lambda/W} \exp\left[ik_y^{(a,\text{L})}y_p\right]$ needs to be added.

Let $E_z^{(a),p}(x,y)$ with $0 \le y \le \Lambda$ be the total field from simulation of the $p$-th unit cell with incident angle $\theta_a$. Then, LPA II simply stitches together such unit-cell field profiles to approximate $E_z^{(a)}(x=L,y)$, as

$$E_z^{(a)\text{LPA-II}}(x=L,y) = \sqrt{\frac{\Lambda}{W}} \sum_{p=1}^{N_p} e^{ik_y^{(a,\text{L})}y_p} E_z^{(a),p}(x=L, y-y_p)\Pi_p(y), \tag{46}$$

where $N_p = 4{,}178$ is the unit of unit cells, and $\Pi_p(y)$ is a rectangular function that equals 1 when $y_p \le y \le y_p + \Lambda$, 0 elsewhere. LPA II includes all propagating and evanescent components of the unit-cell simulations, all of which are contained in $E_z^{(a),p}(x,y)$.

Oftentimes, only the zeroth-order (*i.e.*, $a = b = 0$) transmission coefficient of the unit cell is considered. Therefore, LPA I uses

$$E_z^{(a)\text{LPA-I}}(x=L,y) = \sqrt{\frac{\Lambda}{W}} \sum_{p=1}^{N_p} e^{ik_y^{(a,\text{L})}y_p} E_z^{(a),p;\text{prop}}(x=L, y-y_p)\Pi_p(y), \tag{47}$$

where

$$E_z^{(a),p;\text{prop}}(x=L,y) = t_p \frac{e^{ik_y^{(a,\text{R})}y}}{\sqrt{\Lambda k_x^{(a,\text{R})}}}, \quad 0 \le y \le \Lambda \tag{48}$$

is the zeroth-order propagating component of the $p$-th unit cell as in Eq. (7), with $t_p$ being the corresponding transmission coefficient. Since $\Lambda < \lambda/2$ here, this zeroth-order component is the only propagating component on the transmitted side.

Given the approximate $E_z^{(a)}(x=L,y)$, we then use the same angular spectrum propagation procedure to obtain the approximate $E_z^{(a)}(x=L+f,y)$.

## 15. APF-C COMPRESSION ERROR

Here, we characterize the APF-c compression error of the metalens systems. As described in the main text, we compute the relative $\ell^2$-norm error $\|\mathbf{I}-\mathbf{I}_0\|_2/\|\mathbf{I}_0\|_2$, with $\mathbf{I}_0$ being a vector containing the intensity $|E_z^{(a)}(x=L+f,y)|^2$ at the focal plane within $|y| < W/2$ calculated from APF without compression, and $\mathbf{I}$ from APF-c. **Supplementary Fig. 10** plots the error as a function of the incident angle for varying numbers of padded channels ($M_{\text{pad}}$) and varying truncation window widths ($w_{\text{t}}$), with and without the Hann window scaling. We can see that the Hann window significantly reduces the compression error when channel padding is used. By increasing $M_{\text{pad}}$ and $w_{\text{t}}$, the APF-c compression error can be reduced to arbitrarily small. The choice used for Figs. 4–5 in the main text corresponds to the red curves here, with $M_{\text{pad}} = 2{,}000$ (which is about half of the channel numbers $M = 3{,}761$ and $M' = 3{,}841$) and $w_{\text{t}} = 10\lambda$. The same $w_{\text{t}}$ and $M_{\text{pad}}$ are used on both the left (incident) and the right (transmitted) sides.

## 16. DISCRETIZATION ERROR

The grid size $\Delta x$ in the finite-difference discretization and the number of Fourier components in RCWA are chosen to ensure sufficient accuracy, which we describe here.

For metalenses, the most important property is the transmission phase shift. **Supplementary Fig. 11a** shows the discretization error of the zeroth-order transmission phase shift, $\Phi_{\text{FDFD}}^{\Delta x=\lambda/40} - \Phi_{\text{FDFD}}^{\Delta x=\lambda/240}$, of the unit cells described in Sec. 10. We see that the discretization error is negligible away from the resonances, and the error at the resonances arises because the angle at which the resonance exists is highly sensitive on the structure. Here, the wrapped $|\Phi_{\text{FDFD}}^{\Delta x=\lambda/40} - \Phi_{\text{FDFD}}^{\Delta x=\lambda/240}|$ averaged over angles and ridge widths is 0.11 radian. So, we use $\Delta x = \lambda/40$ for the metasurface simulations using APF and using MaxwellFDFD.

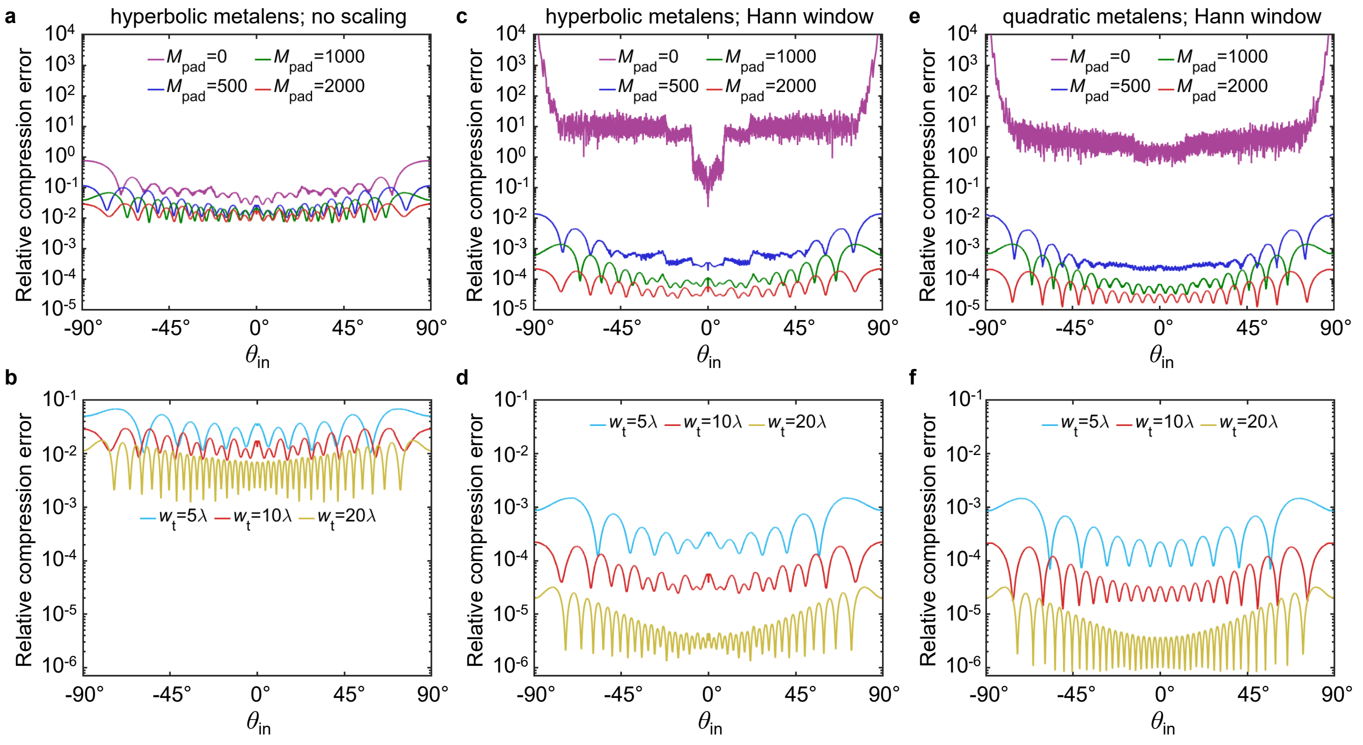


**Supplementary Fig. 10. Compression error of APF-c. a–b**, Angle dependence of the relative compression error for different numbers of padded channels $M_{\mathrm{pad}}$ and different truncation window widths $w_{\mathrm{t}}$, for the mm-wide hyperbolic metalens without Hann window scaling. **c–d**, Same as **a–b** but with the Hann window scaling. **e–f**, Same as **c–d** but for the quadratic metalens. The top row has $w_{\mathrm{t}}$ fixed at $10\lambda$. The bottom row has $M_{\mathrm{pad}}$ fixed at 2,000.

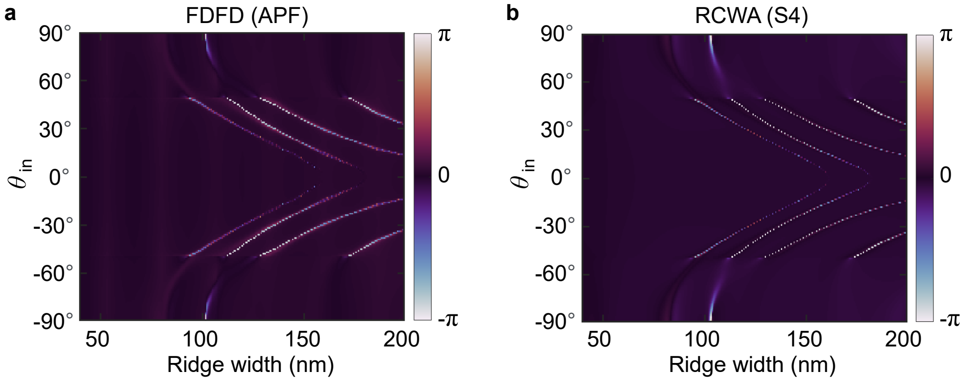


**Supplementary Fig. 11. Discretization error**. Discretization error of (**a**) FDFD, characterized by $\Phi_{\mathrm{FDFD}}^{\Delta x=\lambda/40} - \Phi_{\mathrm{FDFD}}^{\Delta x=\lambda/240}$, and (**b**) RCWA, characterized by $\Phi_{\mathrm{RCWA}}^{\mathrm{numG}=5} - \Phi_{\mathrm{RCWA}}^{\mathrm{numG}=99}$, for the meta-atom zeroth-order transmission phase shifts.

Similarly, **Supplementary Fig. 11b** shows the discretization error of the transmission phase shift for RCWA, $\Phi_{\mathrm{RCWA}}^{\mathrm{numG}=5} - \Phi_{\mathrm{RCWA}}^{\mathrm{numG}=99}$, where numG is the number of Fourier components used in the unit cell simulations. The error is comparable to **Supplementary Fig. 11a**, with averaged $|\Phi_{\mathrm{RCWA}}^{\mathrm{numG}=5} - \Phi_{\mathrm{RCWA}}^{\mathrm{numG}=99}|$ being 0.10 radian. So, we use 5 Fourier components per unit cell (11 Fourier components per $\lambda$) for the metasurface benchmarks with RCWA.

We checked that FDFD and RCWA give consistent results, with $|\Phi_{\mathrm{FDFD}}^{\Delta x=\lambda/240} - \Phi_{\mathrm{RCWA}}^{\mathrm{numG}=99}|$ averaging to 0.03 radian.

[1] Landau, H. Sampling, data transmission, and the Nyquist rate. *Proceedings of the IEEE* **55**, 1701–1706 (1967).
[2] Carminati, R., Sáenz, J. J., Greffet, J.-J. & Nieto-Vesperinas, M. Reciprocity, unitarity, and time-reversal symmetry of the S matrix of fields containing evanescent components. *Phys. Rev. A* **62**, 012712 (2000).
[3] Oskooi, A. & Johnson, S. G. Electromagnetic wave source conditions. In Taflove, A., Oskooi, A. & Johnson, S. G. (eds.) *Advances in FDTD Computational Electrodynamics: Photonics and Nanotechnology*, chap. 4 (Artech House, 2013).
[4] Rumpf, R. C. Simple implementation of arbitrarily shaped total-field/scattered-field regions in finite difference frequency-domain. *Prog. Electromagn. Res.* **36**, 221–248 (2012).
[5] Fisher, D. S. & Lee, P. A. Relation between conductivity and transmission matrix. *Phys. Rev. B* **23**, 6851–6854 (1981).
[6] Datta, S. *Electronic Transport in Mesoscopic Systems* (Cambridge University Press, 1995).
[7] Wimmer, M. *Quantum transport in nanostructures: From computational concepts to spintronics in graphene and magnetic tunnel junctions*. Ph.D. thesis, Universität Regensburg (2009).
[8] Yee, K. S. Numerical solution of boundary value problems involving Maxwell's equations in isotropic media. *IEEE Trans. Antennas Propag.* **14**, 302–307 (1966).
[9] Farjadpour, A. *et al.* Improving accuracy by subpixel smoothing in the finite-difference time domain. *Opt. Lett.* **31**, 2972–2974 (2006).
[10] Gedney, S. Perfectly matched layer absorbing boundary conditions. In Taflove, A. & Hagness, S. C. (eds.) *Computational Electrodynamics: The Finite-Difference Time-Domain Method*, chap. 7 (Artech House, 2005), 3rd edn.
[11] Chew, W. C. Electromagnetic theory on a lattice. *Journal of Applied Physics* **75**, 4843–4850 (1994).
[12] Amestoy, P. R., Duff, I. S., Koster, J. S. & L'Excellent, J.-Y. A fully asynchronous multifrontal solver using distributed dynamic scheduling. *SIAM J. Matrix Anal. Appl.* **23**, 15–41 (2001).
[13] Sayood, K. *Introduction to Data Compression* (Morgan Kaufmann, 2017), 5 edn.
[14] Torfeh, M. & Arbabi, A. Modeling metasurfaces using discrete-space impulse response technique. *ACS Photon.* **7**, 941–950 (2020).
[15] Skarda, J. *et al.* Low-overhead distribution strategy for simulation and optimization of large-area metasurfaces. *NPJ Comput. Mater.* **8**, 78 (2022).
[16] Frigo, M. & Johnson, S. The design and implementation of FFTW3. *Proceedings of the IEEE* **93**, 216–231 (2005).
[17] Smith, S. W. *The Scientist and Engineer's Guide to Digital Signal Processing* (California Technical Pub., 1997), 1 edn.
[18] MESTI. `https://github.com/complexphoton/MESTI.m`.
[19] Arioli, M., Demmel, J. & Duff, I. S. Solving sparse linear systems with sparse backward error. *SIAM Journal on Matrix Analysis and Applications* **10**, 165–190 (1989).
[20] Aieta, F. *et al.* Aberration-free ultrathin flat lenses and axicons at telecom wavelengths based on plasmonic metasurfaces. *Nano Lett.* **12**, 4932–4936 (2012).
[21] Aieta, F., Genevet, P., Kats, M. & Capasso, F. Aberrations of flat lenses and aplanatic metasurfaces. *Opt. Express* **21**, 31530–31539 (2013).
[22] Pu, M., Li, X., Guo, Y., Ma, X. & Luo, X. Nanoapertures with ordered rotations: symmetry transformation and wide-angle flat lensing. *Opt. Express* **25**, 31471–31477 (2017).
[23] Lassalle, E. *et al.* Imaging properties of large field-of-view quadratic metalenses and their applications to fingerprint detection. *ACS Photon.* **8**, 1457–1468 (2021).
[24] Goodman, J. W. *Introduction to Fourier Optics* (W. H. Freeman, 2017), 4 edn.
[25] Cubillos, M. & Jimenez, E. Diffraction integral computation using sinc approximation. *Appl. Numer. Math.* **178**, 69–83 (2022).
[26] Pestourie, R. *et al.* Inverse design of large-area metasurfaces. *Opt. Express* **26**, 33732–33747 (2018).
